# Age-related differences in intramuscular fat distribution: spatial quantification in human ankle plantar flexors

**DOI:** 10.3389/fbioe.2025.1594557

**Published:** 2025-06-02

**Authors:** Zhenkai Zhao, Fiona Elizabeth Smith, Taylor J. M. Dick, Emma Hodson-Tole

**Affiliations:** ^1^ Department of Life Sciences, Manchester Metropolitan University, Manchester, United Kingdom; ^2^ Manchester Institute of Sport, Manchester Metropolitan University, Manchester, United Kingdom; ^3^ Department of Sport and Exercise Sciences, Manchester Metropolitan University, Manchester, United Kingdom; ^4^ School of Biomedical Sciences, The University of Queensland, Brisbane, QLD, Australia

**Keywords:** intramuscular fat, spatial distribution, region-growing method, ankle plantar flexors muscle, delaunay tessellation, clustering

## Abstract

**Indroduction:**

Accumulation of intramuscular fat (IMF) is an important marker of skeletal muscle health, typically reported as the mean intramuscular fat fraction (FF) from quantitative magnetic resonance imaging (MRI). However, such a summary measure does not reveal the spatial distribution of the FF through the muscle volume, and currently no methods to quantify intramuscular FF spatial distribution have been reported. This study assessed two- and three-dimensional characteristics of intramuscular FF spatial distribution and investigated age-related differences in intramuscular FF clustering in medial gastrocnemius (MG), lateral gastrocnemius (LG), and soleus (SOL) muscles.

**Methods:**

A total of 32 physically active young (*N* = 19, 23.8 ± 2.2 years) and older (*N* = 13, 70.1 ± 2.2 years) participants were recruited. Intramuscular FF regions were extracted from axial mDixon MRIs using a region-growing method, revealing branch-like clusters, potentially following the vasculature. Three-dimensional intramuscular FF clustering and density were assessed using Delaunay tessellation and Ripley’s functions.

**Results:**

Older adults exhibited significantly shorter Delaunay mean edge lengths compared to young (MG: 2.6 ± 0.5 mm vs. 3.2±0.4 mm, *p* < 0.001; LG: 2.5 ± 0.6 mm vs. 3.3 ± 0.8 mm, *p* < 0.001; SOL: 2.4 ± 0.4 mm vs. 3.5 ± 0.7 mm, *p* < 0.001), indicating denser clustering. Ripley’s K function confirmed greater clustering in older adults. Two-way ANOVA revealed aging (F statistics = 21, *p* < 0.001, Hedge’s *g* = 1.8) but not sex (F statistics = 1.5, *p* = 0.9, Hedge’s *g* = 0.3) as the main effect for variation in intramuscular FF clustering, with no interaction between these two factors (F statistics = 1.3, *p* = 0.35).

**Discussion:**

This work provides an objective framework for characterizing intramuscular FF spatial distribution, providing a means to track skeletal muscle fatty replacement and provide more robust and sensitive markers of skeletal muscle health.

## 1 Introduction

Limitations in physical function compromise the ability to complete activities of daily living, meaning that many people living with physical disability are unable to live independently (Mitchell et al., 2012). While physical function is multi-faceted, the ability to power all motor functions relies upon the forces generated by skeletal muscles (Mitchell et al., 2012). Muscle size and physiological cross-sectional area (PCSA) are primary determinants of force-generating capacity; however, age-related muscle weakening is attributable to both loss of muscle mass and reduced muscle ‘quality’ (Mitchell et al., 2012) [reduction in force produced per PCSA (Mitchell et al., 2012; Pinel et al., 2021)]. Although the underpinning components of decreased muscle quality are not well defined, the presence of intramuscular fat (IMF) has been strongly implicated.

IMF refers to adipose tissue located within skeletal muscles. In older adults, higher levels of IMF are correlated with decreases in muscle strength, mobility, and impaired metabolic health (Marcus et al., 2010; Addison et al., 2014a). Different patterns of IMF infiltration seem to occur, with susceptibility between muscles potentially influenced by fibre type composition or morphological characteristics (Karapınar et al., 2024). Specific muscles seem more prone to IMF accumulation in conditions such as muscular dystrophy or amyotrophic lateral sclerosis, where fatty infiltration can be an indicator of disease progression and severity (D'Souza et al., 2020; Gorgey and Dudley, 2007; Li et al., 2015; Klickovic et al., 2024). This means IMF measurements have important value as outcome measures in clinical trials (Riem et al., 2024). These findings highlight variation in IMF characteristics across health and disease states. It is therefore important to assess IMF to better understand and manage features of muscle function and metabolic health in healthy and aging populations, and to monitor changes associated with neuromuscular pathologies.

Traditional methods for assessing IMF, such as muscle biopsies, are invasive and provide only localized information of a small region of the muscle. In contrast, advanced imaging techniques, including quantitative magnetic resonance imaging (MRI) and computed tomography (CT), offer non-invasive, detailed, and comprehensive assessments. Specifically in MRI, the concentration of fat in a tissue, or fat fraction (FF), is defined as the signal from the fat protons divided by the total signal from the fat and water protons (Bray et al., 2017). In skeletal muscle, work has predominantly focused on quantifying mean intramuscular FF in a small number of MRI slices either in a few selected muscles (Fischer et al., 2014; Kim et al., 2019) or all visible muscles (Grimm et al., 2018; Reyngoudt et al., 2022; Reyngoudt et al., 2021). However, assessment of a single region of an entire muscle volume can lead to significant under- or overestimates of intramuscular FF levels. For example, Heskamp and colleagues showed greater mean intramuscular FF levels in facioscapulohumeral muscular dystrophy (FSHD) patients tend to initially occur at the distal end of muscles and spread proximally, which, if not quantified, biases assessment of disease progression rate (Heskamp et al., 2022). However, it is unknown if the intramuscular FF distribution observed is a feature of FSHD specifically or one that is a general feature of intramuscular FF accumulation that may, for example, be seen with aging.

While Heskamp and colleagues (Heskamp et al., 2022) highlighted the value of assessing intramuscular FF across the entire muscle length, mean intramuscular FF is a summary measure. Therefore, it does not provide any indication of the spatial distribution of the intramuscular FF within the skeletal muscle such as whether it is densely clumped or more sparsely scattered. Quantifying intramuscular FF spatial properties is important because they could critically influence muscle function. For instance, Rahemi et al. used finite-element modelling to demonstrate that a more sparsely distributed intramuscular FF pattern reduced muscle force compared to clumped, or densely clustered, distribution patterns (Rahemi et al., 2015). However, the patterns of intramuscular FF assessed were not based on any empirical data, as direct objective measures of intramuscular FF distribution patterns have yet to be reported. Beyond some recent maps of intramuscular FF in FSHD patients (Riem et al., 2024), objective measures of intramuscular FF spatial distributions throughout the entire three-dimensional (3D) muscle volume are lacking. Quantifying 3D intramuscular FF distribution in quantitative MRIs is therefore a logical next step to improve understanding of age- and pathology-related muscle weakening and may provide a novel biomarker for monitoring disease progression or assessing therapeutic interventions.

In this study, we use a novel, automated method to quantify intramuscular FF distribution throughout the muscle volume in FF-MRIs. We also determine age-related differences in the distribution of intramuscular FF in the ankle plantar flexors. Specifically, intramuscular FF clusters were identified through the volume of the medial gastrocnemius (MG), lateral gastrocnemius (LG), and soleus (SOL) muscles, with Delaunay tessellations and Ripley’s functions used to quantify spatial dispersion and clustering of the identified clusters. The three muscles were selected as they have different physiological and anatomical characteristics that could influence IMF accumulation and hence intramuscular FF spatial distribution. We hypothesized that, there would be a distal to proximal 2D pattern of decreasing mean intramuscular FF values through the muscle, similar to that seen in FSHD patients (Heskamp et al., 2022). Additionally, we predicted that compared to the MG and LG muscles, the SOL muscle would have significantly different 3D intramuscular FF distributions as it has such different muscle fibre type properties and anatomical complexity (Edgerton et al., 1975). Finally, we predicted older participants would have more clumped, spatially clustered, intramuscular FF distributions compared to younger participants, reflecting age-related changes in muscle composition and function.

## 2 Methodology

Nineteen young (7 females, 12 males; 23.8 ± 3.6 years) and thirteen older (5 females, 8 males; 70.1 ± 2.2 years) healthy and physically active adults were included in the study. All participants provided written informed consent and all experimental protocols were approved by The University of Queensland’s Human Research Ethics Committee (#2013001448).

### 2.1 MR image acquisition and processing

All participant data were acquired with a 3T MRI scanner (Magnetom Prisma, Siemens, Germany) in a supine position. An 18-channel body matrix coil was placed on the participants’ legs, as well as an integrated 32-channel spine coil for image acquisition. T1-weighted and m-Dixon scans were acquired from the participants’ dominant leg. The T1-weighted scan covered the entire length of the shank, extending from the distal end of the femur to the ankle, covering 260 transverse slices. The scan protocol used a 2D turbo spin echo (TSE) with a field of view (FOV) of 262 × 350 mm and an acquisition matrix of 336 × 448, reconstructed to 672 × 896. Repetition time/echo time (TR/TE) was 11.7/5.29 ms with a flip angle of 10°, one signal average (NSA), and a total scan time of 462 s, split into two sequences of 231 s each. The mDixon scans covered 192 transverse slices and focused on the muscle bellies of LG, MG, and SOL. A 2-point 3D multi-echo mDixon fast field echo (FFE) sequence, with a FOV of 284 × 350 mm, an acquisition matrix of 260 × 320, and a slice thickness of 2 mm, without a gap was acquired. The scan parameters were TR/TE1/TE2 = 4.33/1.35/2.58 ms, flip angle = 9°, one NSA, and a total scan time of 97 s (D'Souza et al., 2020; Bolsterlee et al., 2019).

An experienced examiner used a combination of semi-automated (Sashimi Segmentation V1.1 (D'Souza et al., 2020)) and manual segmentation (ITK-SNAP v3.8.0, NIH, United States) to delineate the boundary of the MG, LG, and SOL muscles on all slices of the T1-weighted image, when the respective muscle was visible on the scan. The segmentations from Sashimi were imported into ITK-SNAP for inspection, as the automated tool occasionally misclassified boundaries in regions of low contrast or high intramuscular FF. Manual adjustments were made as needed to ensure anatomical accuracy. Following this, a 3D reconstruction was performed in ITK-SNAP to visually assess the combined segmentations of the three muscles. In regions where muscle boundaries were directly adjacent, fat voxels located at the interface were assigned to the muscle whose segmentation label enclosed the voxel. As segmentation masks were manually corrected and non-overlapping, each fat voxel was uniquely assigned to a single muscle region.

### 2.2 Intramuscular fat fraction quantification

#### 2.2.1 Region growing method to extract intramuscular fat fraction clusters

To quantify intramuscular FF from the FF map (computed from the m-Dixon image using the fat-to-total signal ratio), the MG, LG, and SOL were studied separately, using a custom-built MATLAB (MathWorks, Natick, MA, United States) tool. Each muscle segmentation was eroded by 2 pixels to exclude blood vessels and defined the region of interest (ROI), ${FF}_{\text{ROI}}\left(x,y\right)$ , where $\left(x,y\right)$ are the pixel coordinates of the FF value. The median FF, ${FF}_{\text{median}}$ , was calculated from these values for each slice. Given the studied participants were healthy individuals, we expect ${FF}_{\text{median}}$ to be low, such that pixels with an intensity close to this value would represent voxels predominantly comprising muscle tissue. As the approach assumed that muscle tissue remains the dominant component of the muscle volume in the physically active participants, a slice-dependent ${FF}_{\text{median}}$ was considered to provide a reasonable estimate of the local muscle signal at each slice along the muscle length. The thresholding would enable the major intramuscular FF components to be isolated in subsequent analysis steps. The potential influence of this thresholding was evaluated and is reported in Supplementary File S1.

In the region growing process, the region was initiated by selecting a seed point $\left(x_{s},y_{s}\right)$ within the muscle ROI, defined as the pixel with the minimum intramuscular FF difference from ${FF}_{\text{median}}$ : $\left(x_{s},y_{s}\right)=\arg\min_{\left(x,y\right)}\Delta FF\left(x,y\right)$ . The region grows by including neighboring pixels $\left(x_{n},y_{n}\right)$ that satisfy the condition $\Delta FF\left(x_{n},y_{n}\right)\leq {FF}_{median}$ . The search for neighboring pixels occurred across a radius r , defined here as 20 pixels used for all the participants to ensure that regions of muscle tissue surrounded by IMF would be included, with the search able to pass through areas with relatively high intramuscular FF values, which could exist in older participants. Sensitivity analysis of this radius selection was completed (see Supplementary File S1), and for radii ranging from 5 to 30 voxels, no differences were found for intramuscular FF-related metrics.

The mask of the muscle ROI was then updated by excluding all pixels that were predominantly assigned to muscle tissue. We term this the intramuscular FF mask. To remove individual pixels or very small pixel groups, which likely represented imaging artifacts (Wong et al., 2020), the intramuscular FF mask was further refined to ensure only genuine intramuscular FF clusters were retained for further analysis. An empirical approach (visual inspections across multiple participants and muscles) was used to determine a threshold of four connected voxels to be the minimum number that defined a “small cluster.” As four voxels comprised <0.01% of the total voxels of muscle volume, this approach balanced noise elimination with the preservation of biologically meaningful, spatially coherent, intramuscular FF regions.

To quantify the 2D distribution of intramuscular FF values along the muscle length, two values were calculated: 1) the mean intramuscular FF, calculated as the average intramuscular FF value within each muscle ROI (eroded segmentation), providing an indication of IMF magnitude; 2) the intramuscular FF pixel percentage (%), calculated as the ratio of the number of pixels in the intramuscular FF mask to the total number of pixels in the muscle ROI of the corresponding image slice, indicating the proportion of the muscle in which intramuscular FF clusters were identified.

As muscle length and, hence, the number of segmented slices differed between muscles and participants, slice numbers were converted to percentage muscle length, with 0% representing the distal muscle and 100% the proximal muscle. To assess the spatial distribution and density variations in mean intramuscular FF and intramuscular FF pixel percentage along the muscle length, contours of each value, plotted as function of muscle length, were defined by applying an adaptive bandwidth for each curve quantified based on Scott’s rule (Yoshiko et al., 2017).

#### 2.2.2 Spatial analysis of intramuscular fat fraction using delaunay tessellations and Ripley’s functions

Delaunay tessellations connect intramuscular FF voxels into tetrahedron. Measuring the tetrahedron edge lengths and volumes quantifies clustering or local point density. To ensure the measurements were not affected by differences in muscle volume, all metrics were normalized to the corresponding muscle’s volume. Ripley’s K-function provides an indication of whether points can be considered clustered, dispersed or to be completely randomly distributed. The assessment is based on the average number of neighbouring points for each point of interest, calculated across different scales (distance from the given point of interest). The resulting values are compared to the values expected for complete spatial randomness (CSR), with the distance at which values cross the CSR line indicating the distance over which clustering can be considered to occur. To aid interpretability, the L function, defined as $L\left(r\right)=\sqrt{K\left(r\right)/\pi }$ , is included, where deviation above or below the CSR line more clearly indicate clustering or dispersion, respectively.

### 2.3 Statistical analysis

All statistical analyses were performed using Python’s SciPy and scikit-posthocs, with visualization via Matplotlib and Seaborn. Pearson’s correlation coefficient assessed the relationship between mean FF and FF pixel percentage along the muscle length. Normality and homogeneity of variances of the Delaunay mean edge lengths and volumes of each muscle were tested using the Shapiro-Wilk and Levene’s tests. If assumptions were met, a two-way ANOVA (p < 0.05) assessed age and sex effects, followed by Tukey’s HSD for *post hoc* comparisons. If assumptions were violated, the Friedman test was used, followed by the Kruskal–Wallis test and Dunn’s *post hoc* test with Bonferroni correction. Considering the small sample size, effect sizes (Hedges’ g) were assessed to directly quantify the magnitude of differences, allowing interpretation regardless of statistical significance or sample size limitations.

## 3 Results

### 3.1 Two-dimensional fat fraction distribution along the muscle length

**FIGURE 1:**
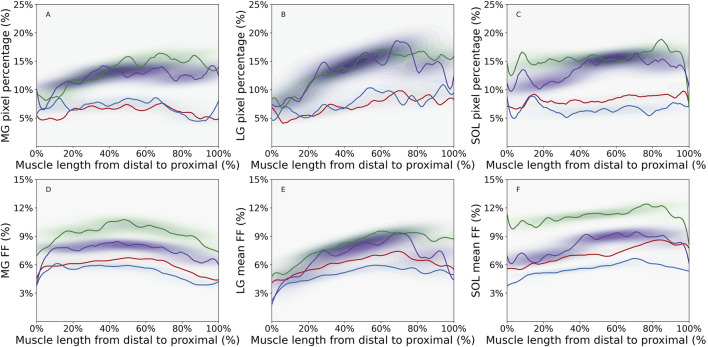
Density plot of intramuscular fat fraction (FF) pixel percentage for **(A)** Medial Gastrocnemius (MG) muscle, **(B)** Lateral Gastrocnemius (LG) muscle, **(C)** Soleus (SOL) muscle and mean intramuscular FF for **(D)** MG muscle, **(E)** LG muscle, and **(F)** SOL muscle, showing the distribution of values along the distal-proximal muscle length for young male (YM) (red line), young female (YF) (blue line), older male (OM) (green line), and older female (OF) (purple line) participant groups. Contours were defined using an adaptive bandwidth approach based on Scott’s rule (Yoshiko et al., 2017). The band width represents the variations of the parameter along the muscle length. The color of the contour represents the probabilities of parameter values at specific locations along the muscle length. For instance, darker contour indicates higher probablities of parameter values in a specific muscle region. The figure was created using Python (Matplotlib) and saved as JPG.

As displayed in Figures 1A–C, the overall intramuscular FF pixel percentages were greater in the older compared to the young adults. In MG, the older male group had the greatest intramuscular FF pixel percentage, with values increasing from distal to proximal regions, and stabilizing as they approached the proximal region. In LG, the largest values were seen in the older female group (Figure 1B), and in general there was a steeper increase in intramuscular FF pixel percentage from distal to proximal regions than seen in MG. In the SOL muscle (Figure 1C), the largest values were observed in the older male group with smaller fluctuations in values across the muscle length observed in each group compared to MG and LG. In MG and LG, the greatest values occurred in the proximal muscle region in most groups except for the older female group (Table 1). In the SOL muscle, the greatest values occurred in the distal region but shifted to the proximal region for older groups.

**TABLE 1:** Peak values for intramuscular fat fraction (FF) pixel percentage and mean intramuscular FF along the muscle length with their locations for Medial Gastrocnemius (MG), Lateral Gastrocnemius (LG), and Soleus (SOL) muscles of different groups [young male, young female, older male, older female].

Muscle	Group	Peak intramuscular FF pixel percentage	Location (% muscle length)	Peak mean intramuscular fat fraction	Location (% muscle length)
MG	Young Male	7.7%	71%	6.7%	50%
	Young Female	8.7%	17%	6.2%	11%
	Older Male	17.4%	70%	10.8%	48%
	Older Female	15.1%	37%	8.5%	44%
LG	Young Male	10.1%	71%	7.4%	70%
	Young Female	10.5%	92%	6.0%	55%
	Older Male	18.0%	62%	9.8%	62%
	Older Female	19.3%	70%	9.5%	70%
SOL	Young Male	10.8%	17%	8.6%	84%
	Young Female	9.7%	15%	6.7%	67%
	Older Male	19.9%	85%	12.5%	76%
	Older Female	16.8%	71%	9.6%	72%

The mean intramuscular FF distributions were generally similar to the intramuscular FF pixel percentage in the muscles studied, except for the proximal region of MG muscle for older groups and the distal region of SOL muscle for the young female group (Figures 1D–F; Table 1). In the young male group, there was moderate to strong correlations in all three muscles, indicating a consistent relationship between the two parameters (Table 2). Similarly, the young female group also exhibited strong correlations in the MG and LG muscles, but a poor correlation in the SOL muscle, indicating that mean intramuscular FF within specific regions of the muscle does not align consistently with the extent of spatial coverage represented by the intramuscular FF pixel percentage. In older males and females, mean intramuscular FF strongly correlated with intramuscular FF pixel percentage in LG and SOL, but only moderately in MG.

**TABLE 2:** Pearson correlation coefficient for intramuscular fat fraction pixel percentage and mean intramuscular fat fraction for different groups (young male, young female, older male, older female) and muscles [Medial Gastrocnemius (MG), Lateral Gastrocnemius (LG), and Soleus (SOL)].

Group	MG	LG	SOL
Young Male	0.72	0.80	0.67
Young Female	0.80	0.76	−0.02
Older Male	0.46	0.98	0.77
Older Female	0.43	0.94	0.93

The contour bandwidths, visible in Figure 1, are detailed in Table 3. Larger bandwidths indicate greater spatial variability along the muscle. In general, larger values were observed for intramuscular FF pixel percentage compared to mean intramuscular FF in all three muscles in each group. For most of the comparisons between young vs. older age groups, older participants showed the largest bandwidth. The only exception was in the SOL for young males, with a slightly lower value (0.43 vs. 0.36). Interestingly, while the mean intramuscular FF bandwidth values were moderate in size in older participants, the difference in intramuscular FF pixel percentage bandwidth values between young and older groups were larger. In terms of the inter-muscle comparisons, the LG muscle displayed the largest bandwidth, indicating the largest variability of the two parameters along the muscle length.

**TABLE 3:** Adaptive bandwidth for mean intramuscular fat fraction (Mean FF) and intramuscular fat fraction pixel percentage (FFPP) plotted as a function of muscle length for different groups [young male, young female, older male, older female] and muscles [Medial Gastrocnemius (MG), Lateral Gastrocnemius (LG), and Soleus (SOL)]. Greater bandwidth indicates more variabiliity for the metric along the muscle length.

	Young male			Young female			Older male			Older female		
	MG	LG	SOL	MG	LG	SOL	MG	LG	SOL	MG	LG	SOL
Mean FF	0.32	0.40	0.43	0.35	0.36	0.31	0.45	0.67	0.36	0.39	0.79	0.51
FF PP	0.44	0.71	0.39	0.58	0.81	0.47	1.16	1.46	0.76	0.86	1.52	0.94

### 3.2 Three-dimensional spatial distribution of intramuscular fat fraction

**FIGURE 2:**
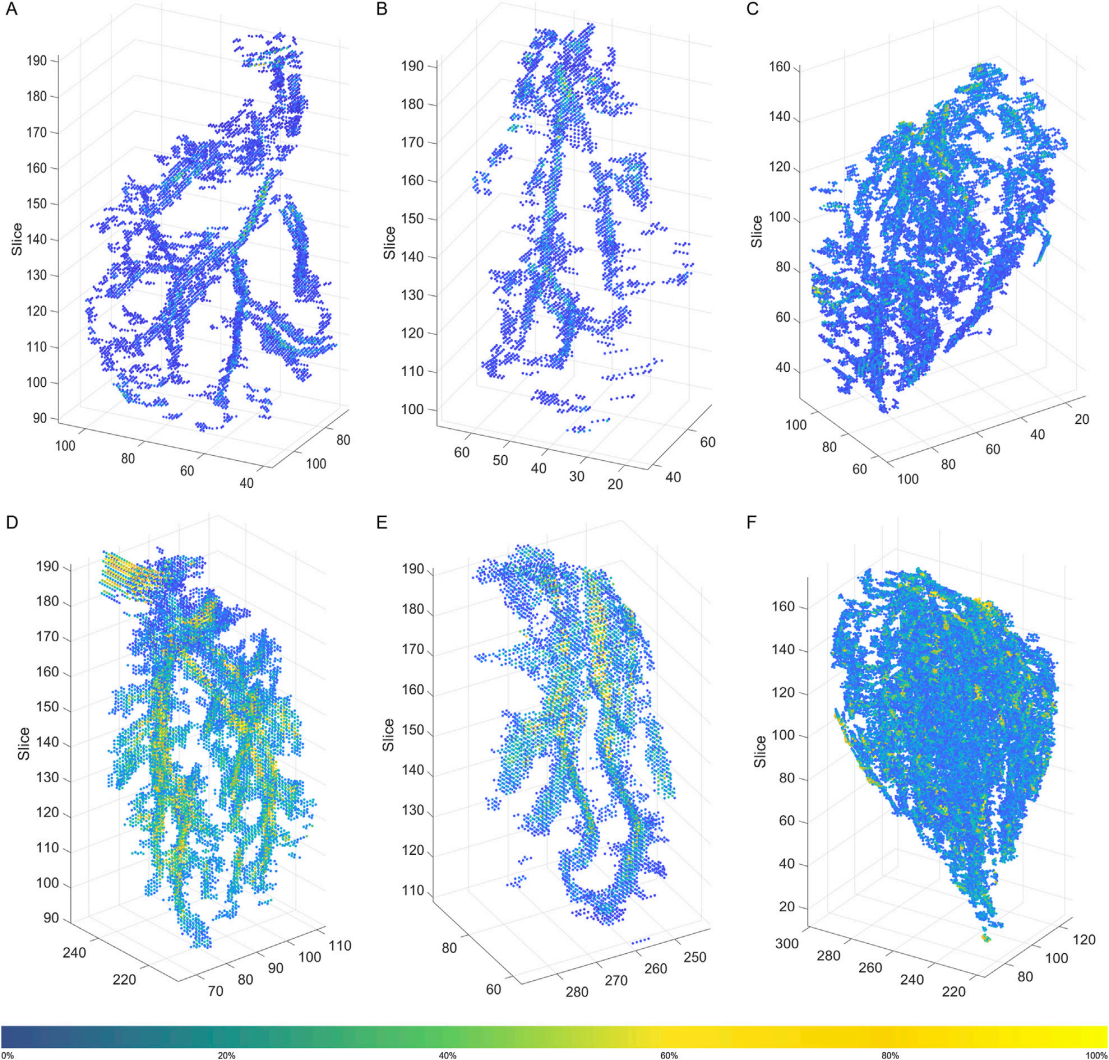
3D distribution of filtered intramuscular fat fraction clusters in a young participant **(A)** Medial Gastrocnemius (MG), **(B)** Lateral Gastrocnemius (LG), and **(C)** Soleus (SOL), and an older participant **(D)** MG, **(E)** LG, and **(F)** SOL. The color bar represents the magnitude of the intramuscular fat fraction (%) for the displayed voxels. The figure was created using Python (Matplotlib) and saved as TIFF.

In general, 3D intramuscular FF distribution was similar in each of the muscles for participants within the same age and sex group (see Supplementary File S2). There were however distinct differences in the patterns observed between the three muscles and the participant groups. In young participants, the MG and LG showed a similar pattern with branch-like structures of intramuscular FF voxels through the muscle volume; compared to the LG muscle, MG showed more branches (Figures 2A,B). For the SOL of the young participants, a broader distribution of intramuscular FF voxels with several distinct high-density clusters were observed (Figure 2C). These clusters were dispersed throughout the muscle volume, indicating multiple focal points of IMF accumulation rather than a single branch-like structure. In the older participant, the intramuscular FF voxels appeared more densely clustered (Figures 2D–F), especially for the MG and SOL muscles (Figures 2D,F). More complex and greater branch-like structures were observed in these two muscles, with more densely packed intramuscular FF voxels appearing to be more widely distributed through the muscle volume.

**FIGURE 3:**
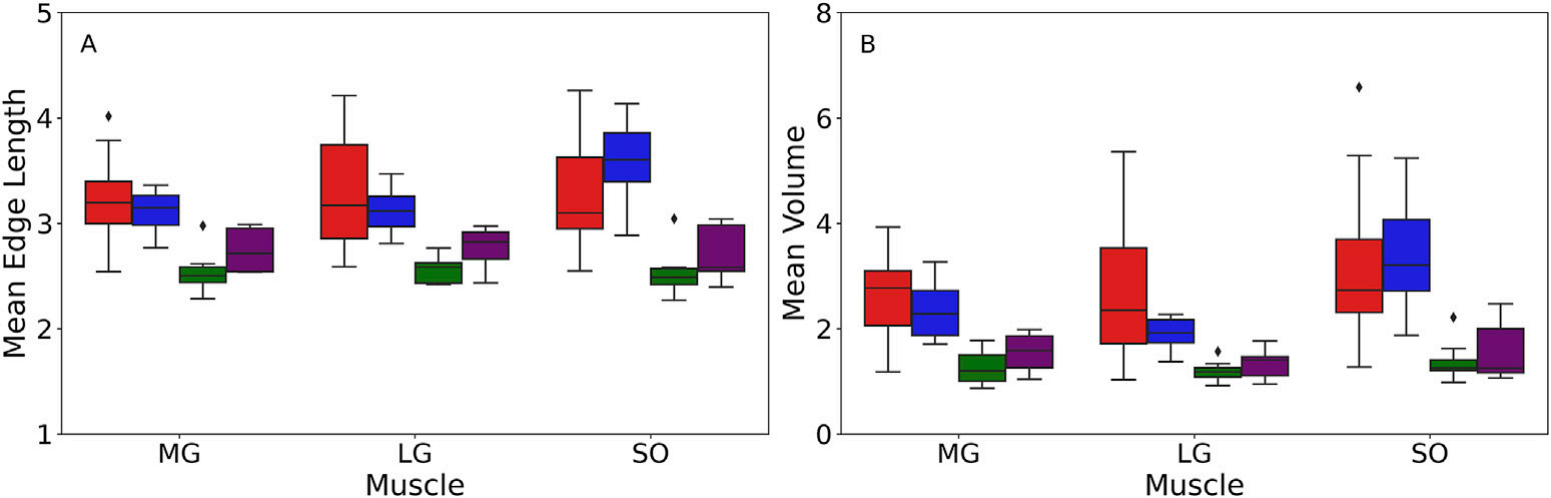
Box plot of the mean edge length measured in mm **(A)** and mean volumes measured in mm^3^ **(B)** acquired from the Delaunay tessellation for different muscles among four groups: young male (YM) (red box), young female (YF) (blue box), older male (OM) (green box), older female (OF) (purple box). The box edge indicates the first (Q1) and third quartiles (Q3), capturing the interquartile range (IQR) of the data. The line within the box represents the median value. The whiskers extend to the minimum and maximum values within 1.5 times the IQR from quartiles. Any points outside the range are displayed as outliers (black dots) and represent values that fall beyond the whiskers. The figure was created using Python (Matplotlib) and saved as TIFF.

Significant main effects of age were observed for both the Delaunay mean edge length and volume within each muscle, with young participants having longer edge lengths than older participants (Figure 3; Table 4), suggesting sparser intramuscular FF distribution with larger gaps between clusters. Specifically, age-only paired comparisons for the mean edge length showed significantly longer values for young vs. older males (MG: mean difference = 0.64 mm, *p* < 0.001, *g* = 1.953; LG: *p* < 0.001, *g* = 1.615) but similar lengths between young and older females (MG: mean difference = 0.33 mm, *p* = 0.2, *g* = 1.475; LG: *p* = 0.75, *g* = 1.416). Only the SOL muscle showed significantly larger values for both males (mean difference = 0.69 mm, *p* = 0.002, *g* = 1.696) and females (mean difference = 0.81 mm, *p* = 0.005, *g* = 2.181). The mean volume paired comparisons revealed the same differences as the mean edge length in males (MG: mean difference = 1.40 mm^3^, *p* < 0.001,*g* 2.056; LG: *p* = 0.002, *g* = 1.396; SOL: *p* = 0.006, *g* = 1.443) and females (MG: mean difference = 0.80 mm^3^, *p* = 0.13, *g* = 1.432; LG: *p* = 0.42, *g* = 1.597; SOL: *p* = 0.04, *g* = 1.721).

**TABLE 4:** Statistics of the Delaunay mean edge length and mean volume for Medial Gastrocnemius (MG), Lateral Gastrocnemius (LG), and Soleus (SOL) for age and sex effects.

Delaunay mean edge length (*F* statistic, *p*-value, Hedge’s *g*)			
Muscle	Age	Sex	Age × sex
MG	26.88	0.016	2.174
	<0.001	0.89	0.15
	1.813	−0.011	
LG	19.30	0.001	1.618
	<0.001	0.98	0.21
	1.552	0.026	
SOL	29.90	2.914	0.191
	<0.001	0.10	0.66
	1.875	−0.414	

Delaunay mean edge length (*F* statistic, *p*-value, Hedge’s *g*)			
MG	28.83	0.076	1.771
	<0.001	0.78	0.19
	1.891	0.093	
LG	14.29	1.552	2.070
	<0.001	0.22	0.16
	1.299	0.383	
SOL	20.06	0.3097	0.001
	<0.001	0.58	0.97
	1.615	−0.136	

Paired age-sex group comparisons showed some significant differences for mean edge length (MG: (young female vs. older male with mean difference = 0.53 mm, *p* = 0.006, *g* = 2.451; young male vs. older female with mean difference = 0.44 mm, *p* = 0.03, *g* = 1.267); LG: young female vs. older male with *p* = 0.01, *g* = 2.737; SOL: young female vs. older male with mean difference = 0.97 mm, *p* < 0.001, *g* = 3.000) and mean volumes (MG: (young female vs. older male with mean difference = 1.09 mm^3^, *p* = 0.008, *g* = 2.239; young male vs. older female with mean difference = 1.11 mm^3^, *p* = 0.009, *g* = 1.479); SOL: young female vs. older male with *p* = 0.004, *g* = 2.291). However, it is important to note that age was the dominant factor for these differences, as the effects of sex and age × sex interaction were not significant in the muscles studied (Table 4). Beyond p-values, effect size analysis using Hedges’ g further demonstrated strong age-related differences in both mean edge length and volume. Across all muscles, Hedges’ g values for age effects in males ranged from 1.40 to 2.06, and in females from 1.43 to 2.18, indicating large effects of age on intramuscular fat distribution patterns. In contrast, sex effects were small to negligible within age groups (Hedges’ g < 0.4 in all cases), and interaction effects remained nonsignificant.

**FIGURE 4:**
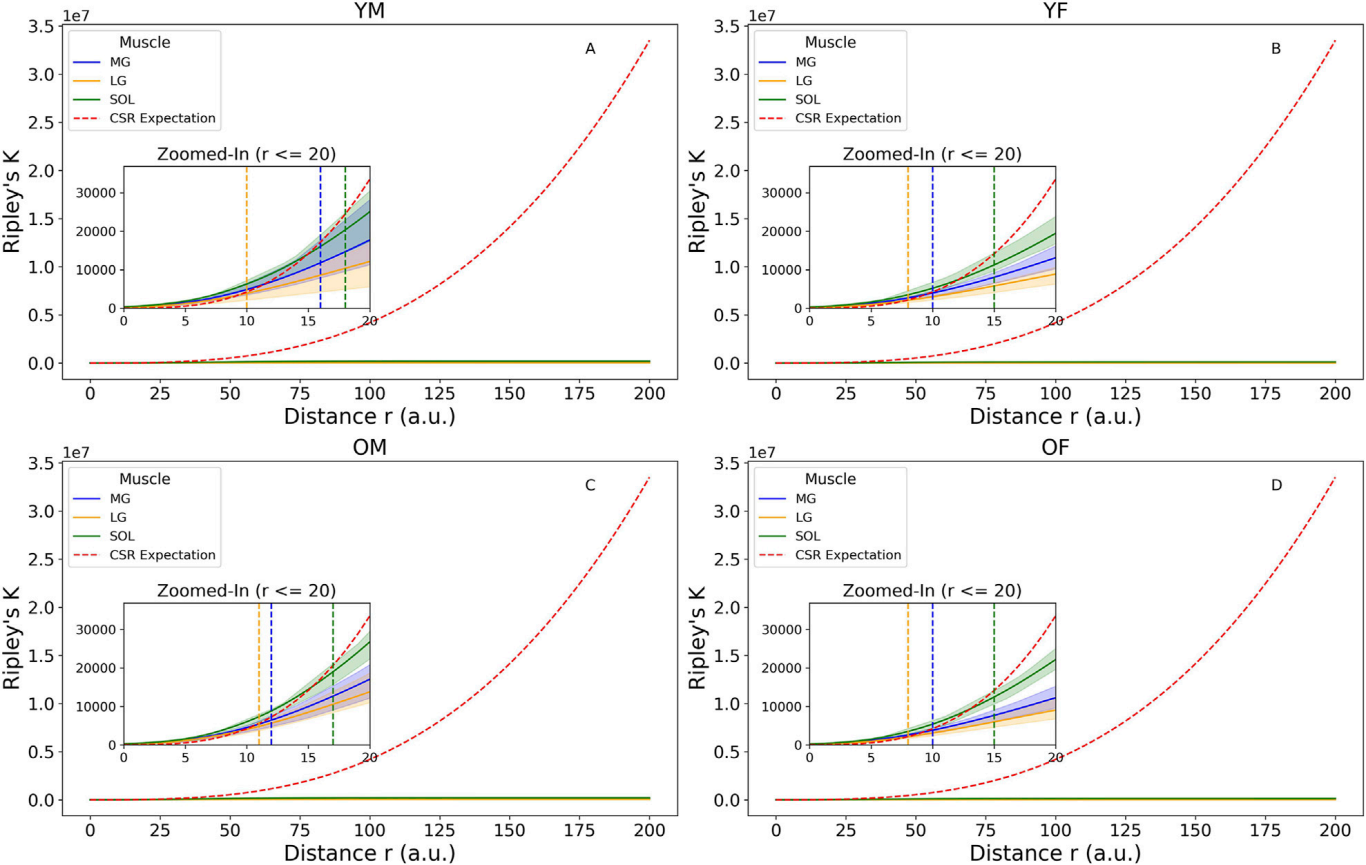
Ripley’s K function with complete spatial randomness (CSR) expectation for four groups: **(A)** young male (YM), **(B)** young female (YF), **(C)** older male (OM), **(D)** older female (OF). Zoomed-in images were plotted to show the distance where the K values are higher than the CSR expectation line, including the 95% confidence envelops to show participant variations. The vertical dotted lines denote the largest distances at which the upper bound of the confidence envelope remain above the CSR line in Medial Gastrocnemius (MG) (blue), Lateral Gastrocnemius (LG) (yellow), and Soleus (SOL) (green). The figure was created using Python (Matplotlib) and saved as JPG.

**FIGURE 5:**
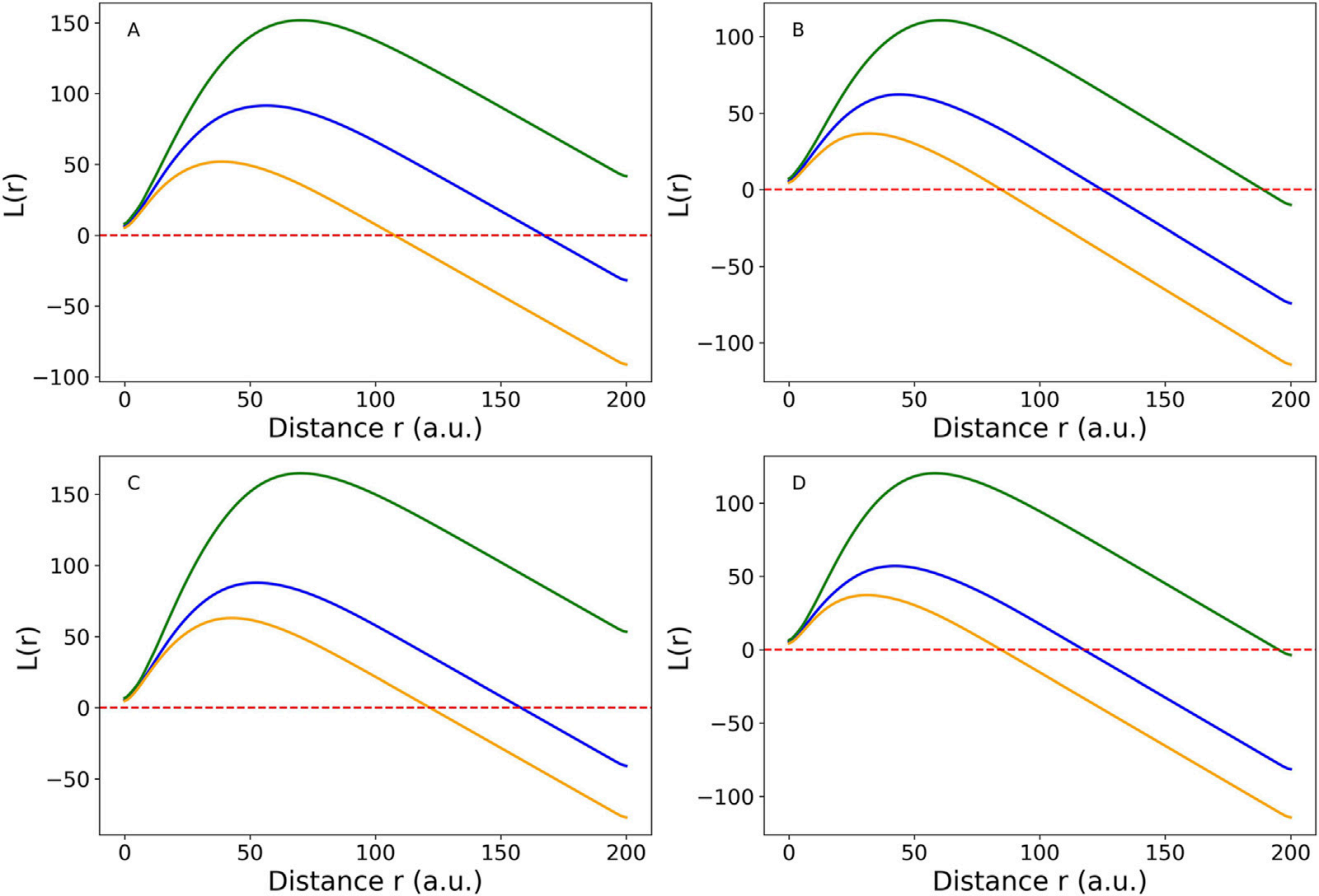
Cross-participant comparison of Medial Gastrocnemius (MG) muscle (blue line), Lateral Gastrocnemius (LG) muscle (orange line), and Soleus (SOL) muscle (green line) using L(r) function, which is a linearized version of Ripley’s K function for four groups: **(A)** young male (YM), **(B)** young female (YF), **(C)** older male (OM), **(D)** older female (OF). The red dotted horizontal line is the complete spatial randomness (CSR) expectation. When the colored lines are above the CSR line, the spatial distribution tends to be clustered. When they are below the CSR line, the spatial distribution is close to random or sparse distribution. The figure was created using Python (Matplotlib) and saved as JPG.

Comparisons between the CSR expectation line and the K values were shown in Figure 4. The largest inter-participant variations occurred in young males. Among all group-muscle combinations, the SOL muscle in the young males exhibited the largest maximum clustering distance, up to 17.5 a.u. This suggests clustering occurs up to this distance, beyond which intramuscular FF clusters become randomly distributed. The inter-muscle comparison showed SOL > MG > LG in clustering strength, a consistent order across participant groups. The L(r) function in Figure 5 provided a transformed view of Ripley’s K function that centers the expectation of CSR at zero. Positive values of L(r) across most distances indicated clustering of intramuscular FF beyond CSR. SOL consistently showed the highest L(r) values, particularly in older adults, indicating stronger and more extended spatial clustering. The older male participants (solid lines) generally exhibited the highest peaks in L(r), indicating dense local intramuscular FF clusters. In contrast, young participants (dotted lines) had lower L(r) values, suggesting more uniform cluster distributions. Notably, in most cases, L(r) values decreased beyond a distance of 75 a.u., approaching or dipping below zero, indicating a transition from clustering to random or even regular distributions beyond that scale.

## 4 Discussion

In this study we used quantitative MRI to provide the first systematic quantification of the two- and three-dimensional spatial distributions of intramuscular FF in the ankle plantar flexors of healthy young and older adults. The intramuscular FF spatial distribution varied between muscles, with denser distributions occurring in SOL (Figures 2, 4). Larger intramuscular FF pixel percentage and mean intramuscular FF were found in older participants, indicating that both the magnitude of IMF and the volume across which it is found increases with age (Figure 1). In MG and LG, larger mean intramuscular FF were found within the proximal muscle region (Table 1), which is inconsistent with previous reports of mean intramuscular FF distribution in patients with FSHD (Heskamp et al., 2022). Taken together, these differences highlight the potential for spatial analysis of intramuscular FF distributions to reveal morphological features of skeletal muscle, which is important for understanding changes in muscle function associated with pathology and aging.

### 4.1 Region growing method for intramuscular fat fraction quantification

The region-growing method incorporates both local intramuscular FF values and their spatial relationships to classify intramuscular FF regions (Rosea et al., 2024). By using spatial connectivity, this method ensures that intramuscular FF voxels are classified in a manner consistent with their neighbouring tissue, providing spatial coherence and reducing the influence of noise or isolated artifacts. Spatially connected voxels that form contiguous clusters are more likely to represent true IMF deposits, as they exhibit similar tissue properties—such as uniform fat content—rather than being isolated voxels that may result from partial volume effects or mixtures of muscle, tendon, and fat (Soret et al., 2007). Additionally, maintaining spatial coherence is a critical advantage over simple thresholding approaches (Maddocks et al., 2014; Young et al., 2015). The method has previously been used to identify intramuscular FF regions (Wong et al., 2020) but was limited to just a few slices rather than the whole muscle volume.

As the region growing algorithm was applied to images from healthy participants, the voxels considered predominantly muscle were identified and removed to leave only FF clusters. This approach may require refinements if applied to clinical data, where the FF values are likely to be much higher and tracking change over time would be valuable. The current method used a data-driven approach to dynamically threshold, resulting in slightly larger values in older participants (See methods). This could have led to smaller IMF masks. However, the greater intramuscular FF pixel percentage values (Figure 1) and more densely clustered intramuscular FF (Figure 2) seen in older adults suggests the differences are unlikely to be an artifact of the threshold values. It should however also be noted that the use of slice-specific ${FF}_{\text{median}}$ as the threshold could lead to inconsistencies in voxel classification across slices. However, ${FF}_{\text{median}}$ reflects the local muscle baseline that can adapt to both anatomical variations and participant-specific differences. A sensitivity analysis with ± 5 variations of ${FF}_{\text{median}}$ threshold was conducted (see Supplementary File S1). Only moderate increases in the total number of intramuscular FF voxels were found with reduced threshold but the spatial distribution remained unchanged (see Supplementary File S1), supporting the robustness of the slice-dependent thresholding method.

### 4.2 Regional variations in the two-dimensional distribution of intramuscular fat fraction

In the MG and LG, and to a smaller extent the SOL, there was an increase in intramuscular FF pixel percentage along the distal-proximal muscle length in the older group, with the distance to values observed in the younger groups also increasing from distal-to-proximal regions (Figure 1). This indicates regional variation in accumulation of IMF may occur because of aging (Pinel et al., 2021). What drives this variation is however unclear. Possible explanations include differences in fibre types along the muscle length. Previous work has suggested that faster fibre types may be preferentially expressed in proximal regions of MG (Hodson-Tole et al., 2013) and it is posited that muscle degeneration, particularly in Type II muscle fibers, leads to increased fat storage (Goodpaster and Wolf, 2004). Additionally, variation in intramuscular pressure distributions during contraction could also influence IMF accumulation, given that adipocytes have been shown to be mechanosensitive (Blade et al., 2024).

We found only two previous reports of variation in mean intramuscular FF levels along the muscle length, both in patient populations. While one study observed a distal-to-proximal decrease (Riem et al., 2024), the other larger study, found more heterogeneous patterns (Heskamp et al., 2022). In contrast, our findings in healthy participants show the highest mean intramuscular FF in proximal muscle regions (Figure 1; Table 1), suggesting different mechanisms may drive IMF infiltration in disease-related *versus* age-related changes. (Addison et al., 2014b). These differences indicate potential for intramuscular FF quantification to distinguish between age- and disease-related changes in muscle characteristics. Therefore, spatial variations in intramuscular FF values could be further exploited to provide more robust and sensitive markers of skeletal muscle health. Such markers would be useful not only as outcome measures in clinical trials but would also have value in diagnosis and monitoring progressive and degenerative diseases to support more personalized decision-making in clinical care.

### 4.3 Variations in three-dimensional spatial distribution of intramuscular fat fraction

We observed branch-like structures for intramuscular FF clusters, however, without direct vascular imaging such as magnetic resonance angiography, this interpretation remains speculative. There was a noticeable difference in the 3D intramuscular FF distribution between the three muscles studied (Figure 2, Supplementary File S2). In MG and LG, clear and large branching structure were observed, while SOL showed much more diffuse intramuscular FF clusters. The differences between muscles may be related to differences in muscle fibre architecture (more variables in SOL muscles with multiple architectural compartments compared to MG and LG muscles) (Edgerton et al., 1975) and/or by differences in fibre type composition: the SOL is predominantly composed of slow-twitch fibers (Gollnick et al., 1974). These fibers have a higher capillary density, allowing them to effectively use oxygen and fatty acids without requiring additional vascular adaptations to support fat storage and metabolism (Saltin and Gollnick, 1983). As a result, FF may accumulate around the capillaries rather than the larger, primary vascular branches. In contrast, MG and LG contain more of a mix of slow- and fast-twitch fibers (Edgerton et al., 1975), and tend to accumulate more extramyocellular lipids (EMCL) especially with aging (Schrauwen-Hinderling et al., 2006). However, a limitation of FF quantification using the mDixon method is that it does not distinguish between intramyocellular lipids (IMCL) and EMCL. EMCL has been shown to exhibit orientation-dependent chemical shift dispersion due to the anisotropic magnetic susceptibility of the surrounding muscle tissue (Khuu et al., 2009). This orientation effect can influence the accuracy of the mDixon method for characterization of intramuscular FF.

## 5 Conclusion

This study provides the first detailed quantification of the 3D distribution of intramuscular FF across the ankle plantar flexors muscles in healthy young and older adults, revealing significant variations across muscles and with aging. The spatial distribution of intramuscular FF can be used to provide valuable insights into the processes of muscle degeneration, with the methods presented providing a means to track distinct processes of skeletal muscle fatty replacement in healthy aging *versus* disease-related conditions. Incorporating clinical data and employing higher-resolution imaging techniques in future research will enhance our understanding of the clinical implications of IMF infiltration, facilitating more targeted interventions and treatment strategies to preserve muscle function and mitigate IMF accumulation in both aging and pathological contexts.

## Data Availability

The raw data supporting the conclusions of this article will be made available by the authors, without undue reservation.
