# Supplementary material for: Age-related differences in intramuscular fat distribution: spatial quantification in human ankle plantar flexors
Source: Front Bioeng Biotechnol. 2025 Jun 2;13:1594557. doi: 10.3389/fbioe.2025.1594557 (PMC12171183; doi:10.3389/fbioe.2025.1594557)
Supplement: Supplementary file 1 [file Table1.docx]

Supplementary Material

**S1.1. Sensitivity analysis for intramuscular fat fraction (FF) threshold and search radius**

**Table S1.1.1.** Sensitivity analysis of intramuscular FF median threshold with ± 5 % variation (sd). Total number of extracted FF voxels for the threshold set to median, median+5% and median-5% were compared. For each group, three participants were randomly selected for analysis. Since the baseline vs +5% does not show differences for the total number of fat voxels, we only include the variations of -5% compared to baseline.

|  | **-5%** | **Baseline** | **+5%** | **Percentage difference**  **(-5% vs baseline)** |
| --- | --- | --- | --- | --- |
| **Young Male** |  | | | |
| MG | 4813 (42) | 3786 (33) | 3781 (44) | 21% |
| LG | 2372 (55) | 1956 (46) | 1956 (40) | 18% |
| SOL | 16504 (113) | 12766 (120) | 12741 (108) | 22% |
| **Young Female** |  | | | |
| MG | 3779 (35) | 3076 (37) | 3056 (30) | 22% |
| LG | 2048 (60) | 1679 (72) | 1678 (66) | 22% |
| SOL | 4259 (88) | 3470 (70) | 3469 (69) | 22% |
| **Older Male** |  | | | |
| MG | 14091 (108) | 12736 (122) | 12736 (101) | 9.6% |
| LG | 7561 (88) | 6798 (72) | 6798 (63) | 10% |
| SOL | 31493 (212) | 28605 (187) | 28600 (199) | 9.1% |
| **Older Female** |  | | | |
| MG | 4496 (40) | 4154 (38) | 4152 (33) | 7.6% |
| LG | 3360 (36) | 3014 (28) | 3012 (29) | 10% |
| SOL | 15090 (144) | 13265 (122) | 13264 (109) | 12% |

**Table S1.1.2** Spatial distribution comparisons for tested intramuscular FF thresholds

| YM | -5% variation | Baseline | +5% variation |
| --- | --- | --- | --- |
| Medial Gastrocnemius | 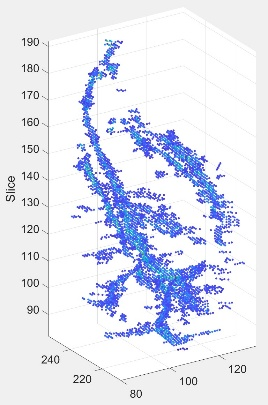 | 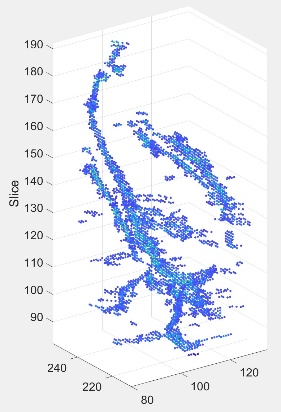 | 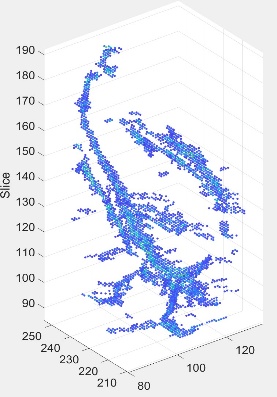 |
| Lateral Gastrocnemius | 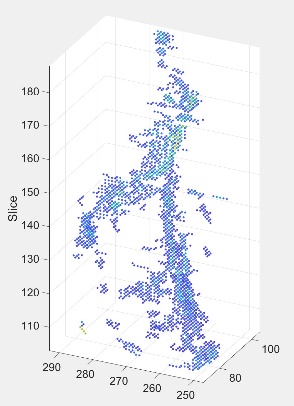 | 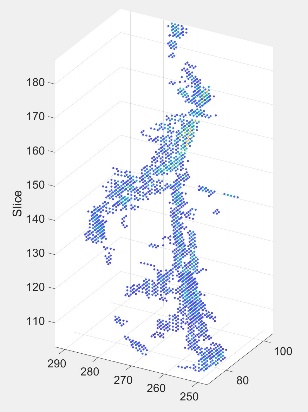 | 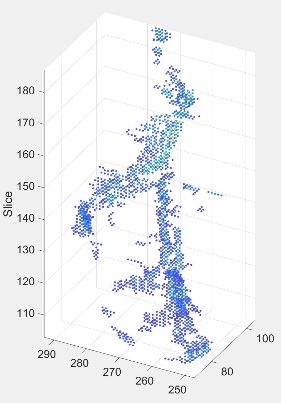 |
| Soleus | 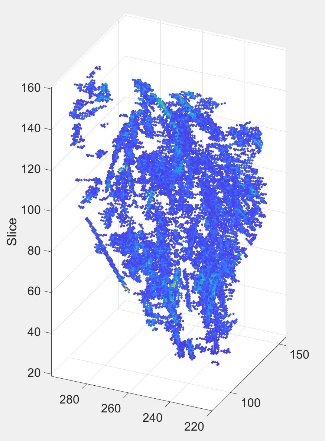 | 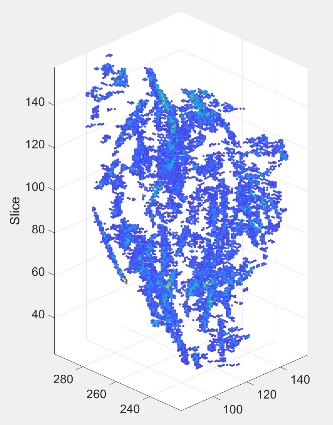 | 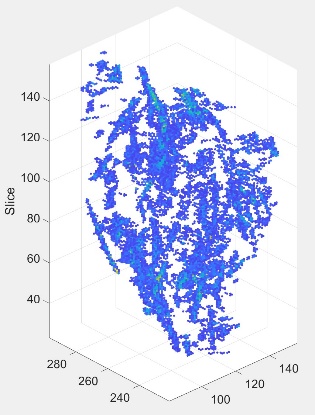 |

| YF | -5% variation | Baseline | +5% variation |
| --- | --- | --- | --- |
| Medial Gastrocnemius | 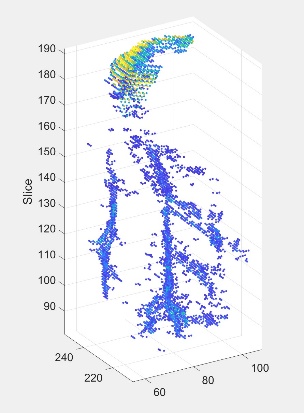 | 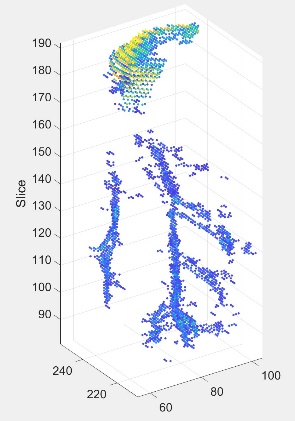 | 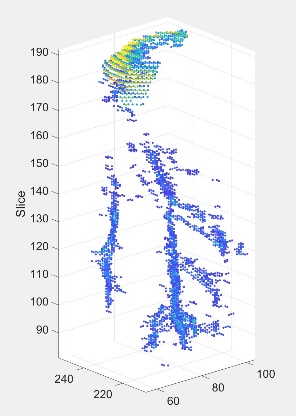 |
| Lateral Gastrocnemius | 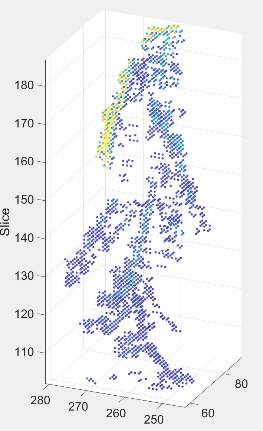 | 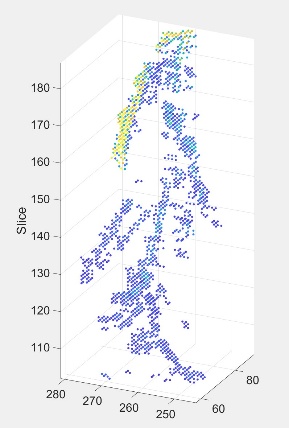 | 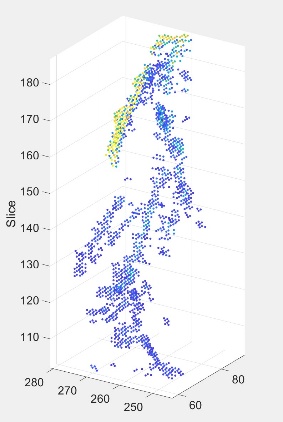 |
| Soleus | 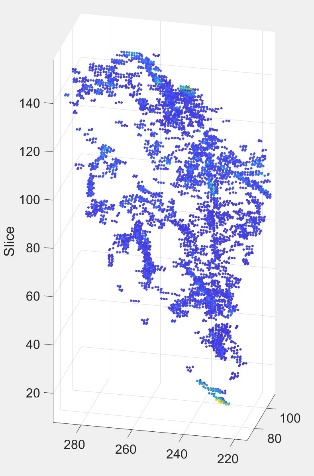 | 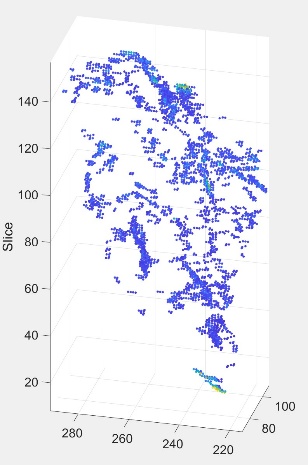 | 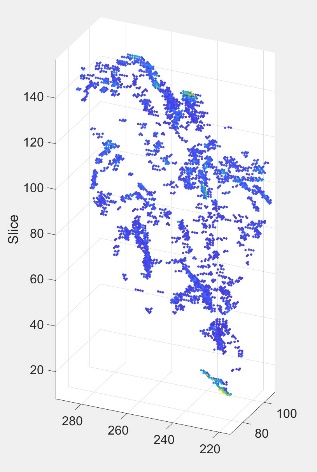 |

| OM | -5% variation | Baseline | +5% variation |
| --- | --- | --- | --- |
| Medial Gastrocnemius | 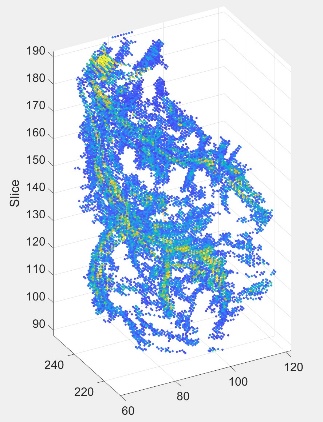 | 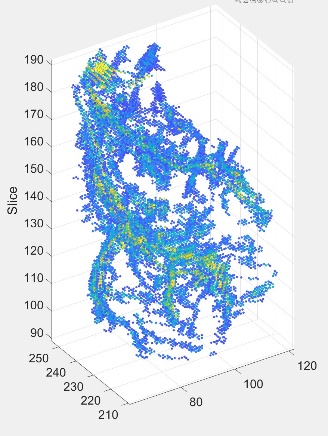 | 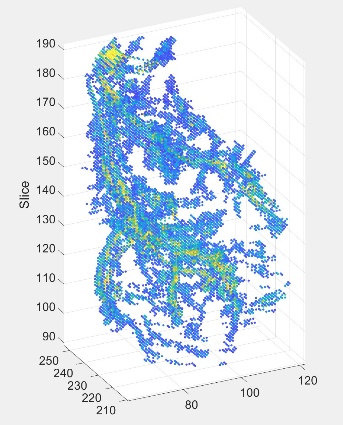 |
| Lateral Gastrocnemius | 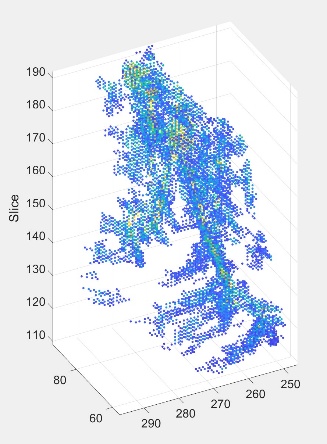 | 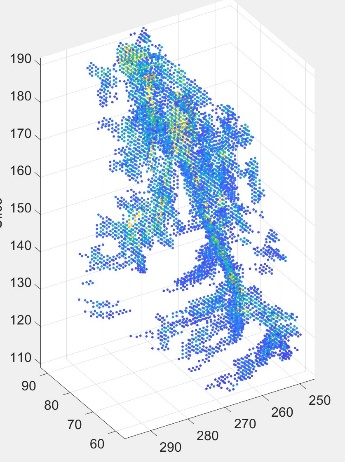 | 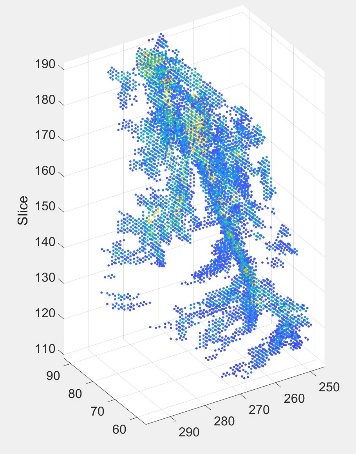 |
| Soleus | 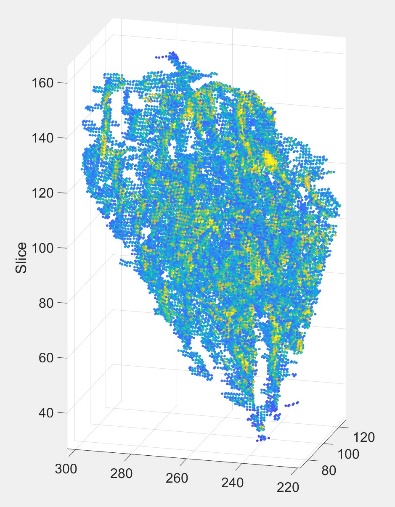 | 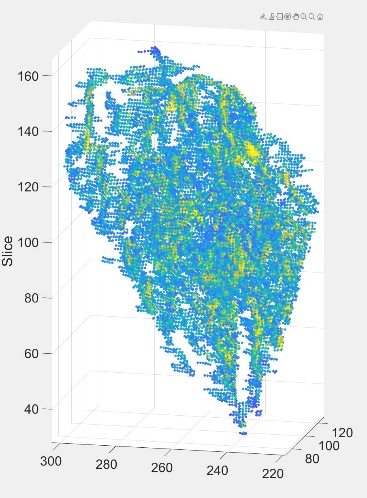 | 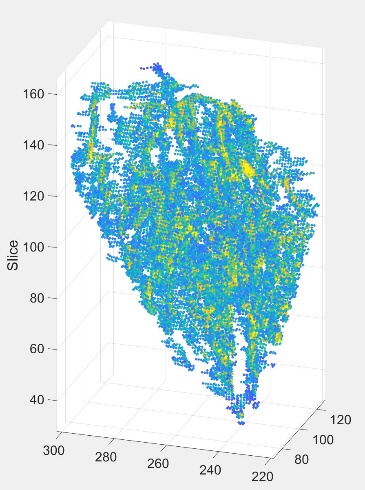 |

| OF | -5% variation | Baseline | +5% variation |
| --- | --- | --- | --- |
| Medial Gastrocnemius | 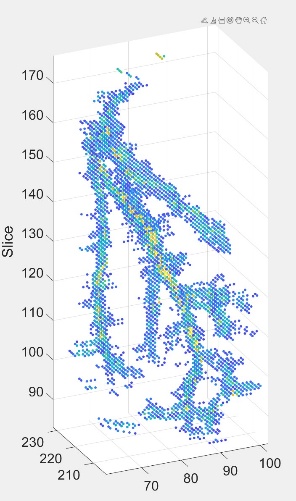 | 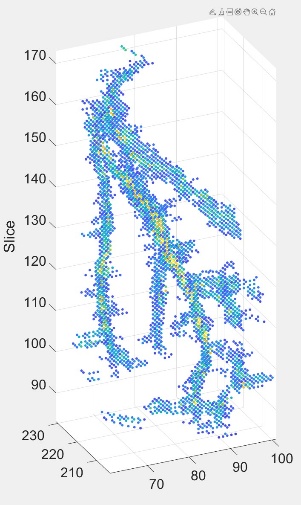 | 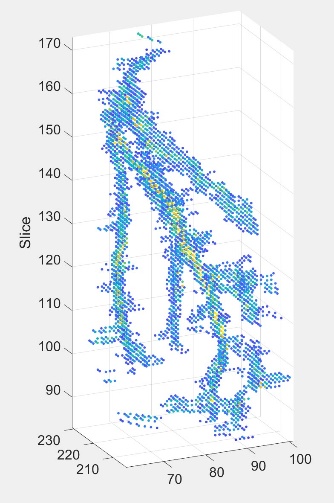 |
| Lateral Gastrocnemius | 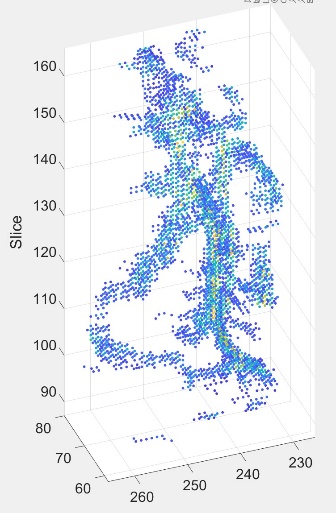 | 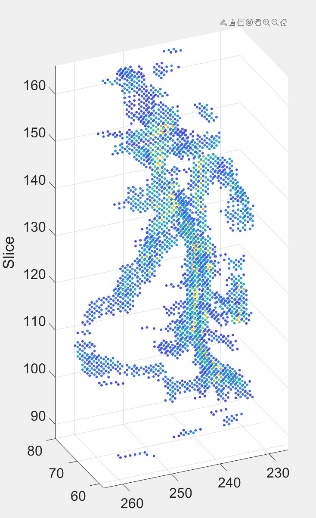 | 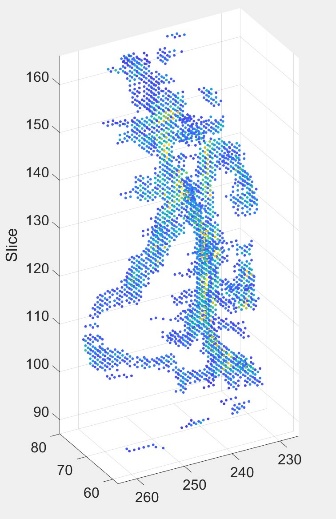 |
| Soleus | 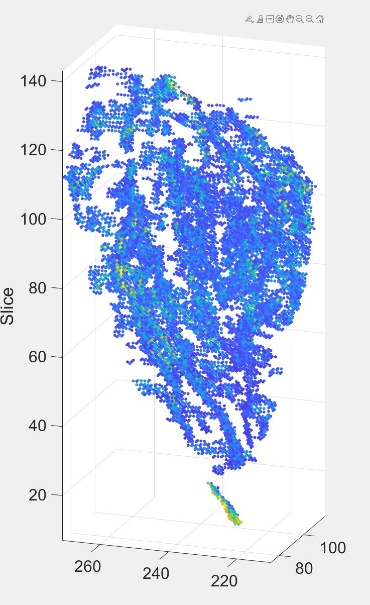 | 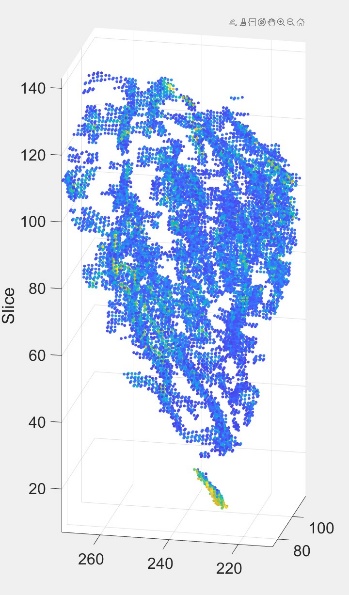 | 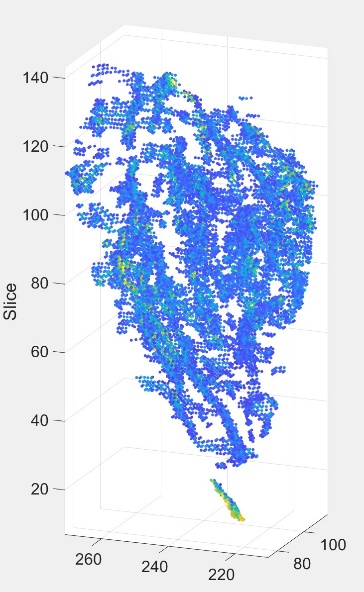 |

**Table S1.1.3.** Sensitivity analysis of searching radius. The searching radius used in the manuscript was 20 voxels. We tested 5, 10, 15, 20, 25, and 30 voxels with the same 12 participants test for threshold sensitivity (above) and compared the total number of intramuscular FF voxels identified.

| Radius (voxels) | 5 | 10 | 15 | 20 | 25 | 30 |
| --- | --- | --- | --- | --- | --- | --- |
| YM | | | | | | |
| MG | 3786 (33) | 3786 (33) | 3786 (33) | 3786 (33) | 3786 (33) | 3786 (33) |
| LG | 1956 (46) | 1956 (46) | 1956 (46) | 1956 (46) | 1956 (46) | 1956 (46) |
| SOL | 12766 (120) | 12766 (120) | 12766 (120) | 12766 (120) | 12766 (120) | 12766 (120) |
| YF | | | | | | |
| MG | 3076 (37) | 3076 (37) | 3076 (37) | 3076 (37) | 3076 (37) | 3076 (37) |
| LG | 1679 (72) | 1679 (72) | 1679 (72) | 1679 (72) | 1679 (72) | 1679 (72) |
| SOL | 3470 (70) | 3470 (70) | 3470 (70) | 3470 (70) | 3470 (70) | 3470 (70) |
| OM | | | | | | |
| MG | 12736 (122) | 12736 (122) | 12736 (122) | 12736 (122) | 12736 (122) | 12736 (122) |
| LG | 6798 (72) | 6798 (72) | 6798 (72) | 6798 (72) | 6798 (72) | 6798 (72) |
| SOL | 28605 (187) | 28605 (187) | 28605 (187) | 28605 (187) | 28605 (187) | 28605 (187) |
| OF | | | | | | |
| MG | 4154 (38) | 4154 (38) | 4154 (38) | 4154 (38) | 4154 (38) | 4154 (38) |
| LG | 3014 (28) | 3014 (28) | 3014 (28) | 3014 (28) | 3014 (28) | 3014 (28) |
| SOL | 13265 (122) | 13265 (122) | 13265 (122) | 13265 (122) | 13265 (122) | 13265 (122) |

Generative AI Disclosure

We confirm that no written or visual content was generated with aI
