# Supplementary material for: Age-related differences in intramuscular fat distribution: spatial quantification in human ankle plantar flexors
Source: Front Bioeng Biotechnol. 2025 Jun 2;13:1594557. doi: 10.3389/fbioe.2025.1594557 (PMC12171183; doi:10.3389/fbioe.2025.1594557)
Supplement: Supplementary file 2 [file Table2.docx]

Supplementary Material

**The figures below show the 3D patterning of all the participants and divided into four ages × sex groups. They were extracted from participants with different dominant legs; therefore, directions of those fat distribution could be different.**

**YM Group (in the order of MG LG and SOL from left to right with participant number on top left)**

S01


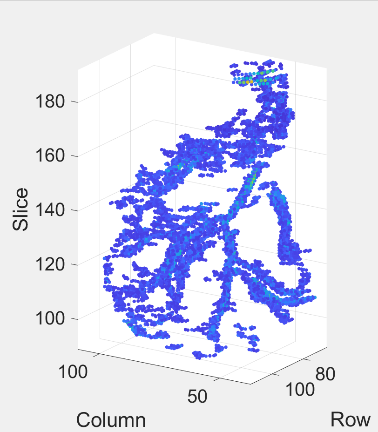

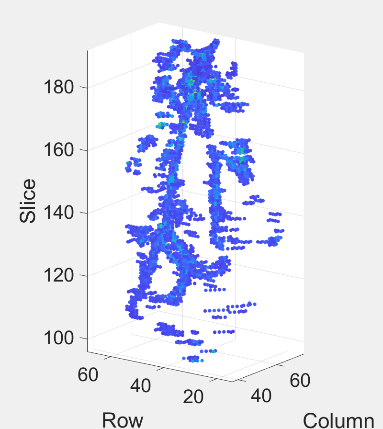

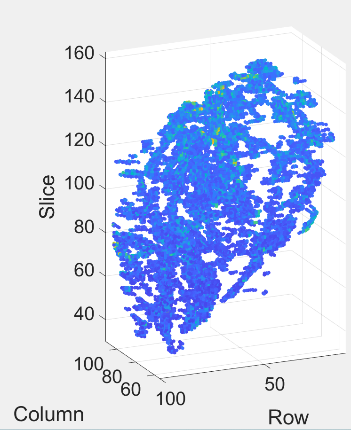

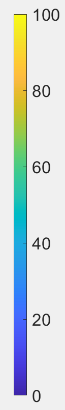


S02


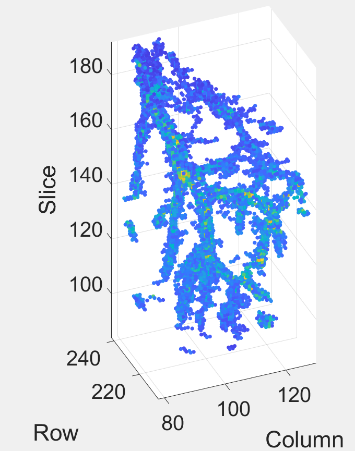

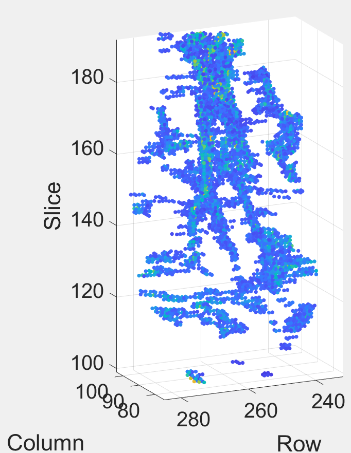

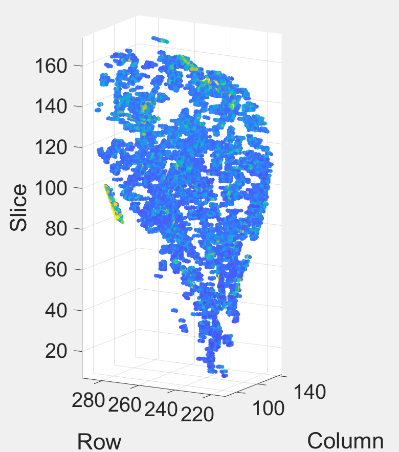

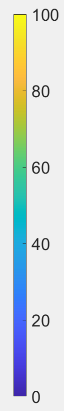


S03


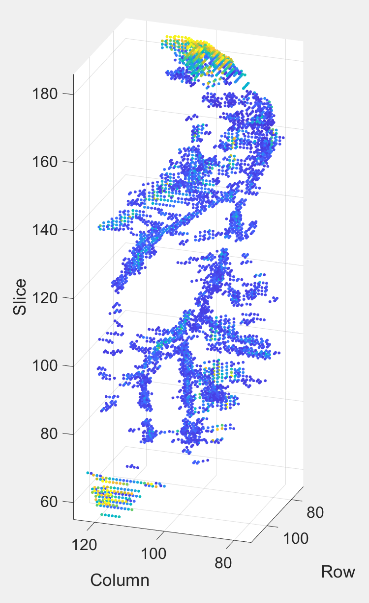

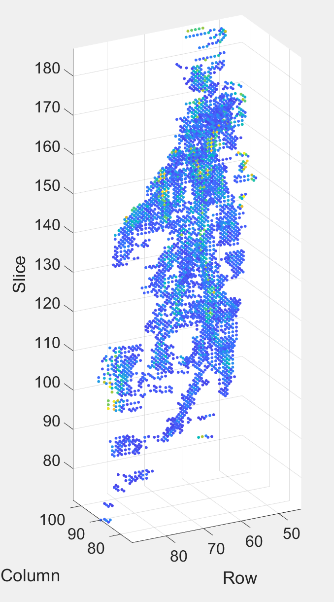

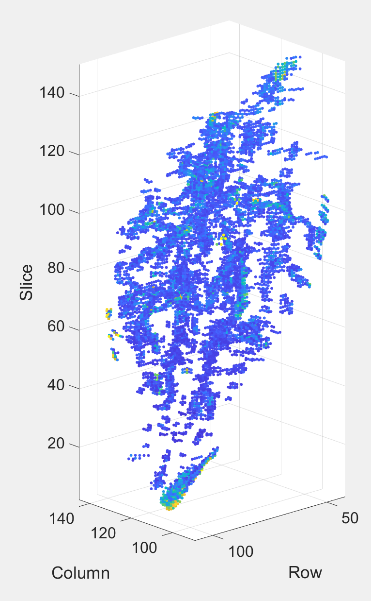

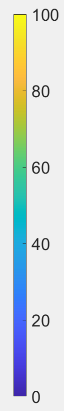


S06


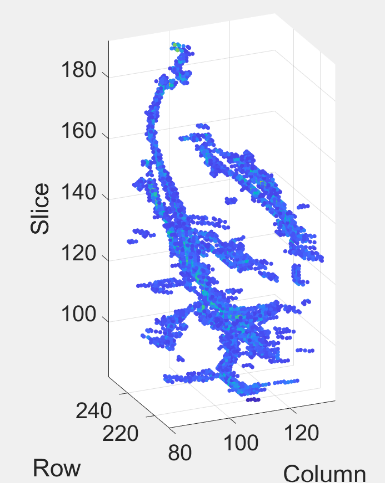

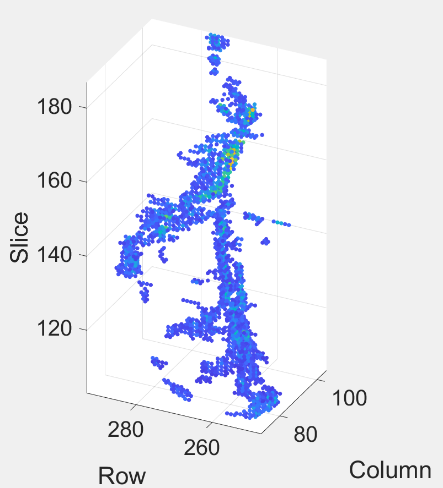

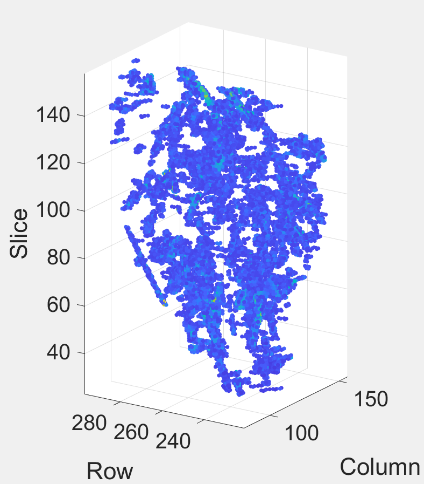

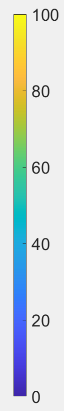


S07


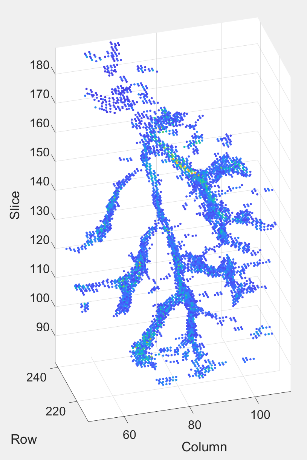

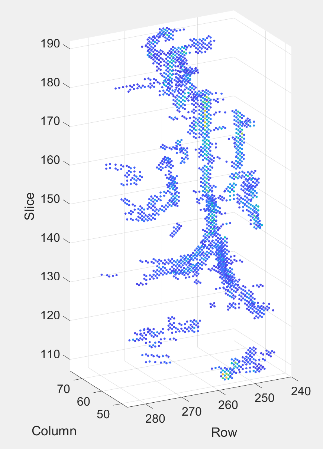

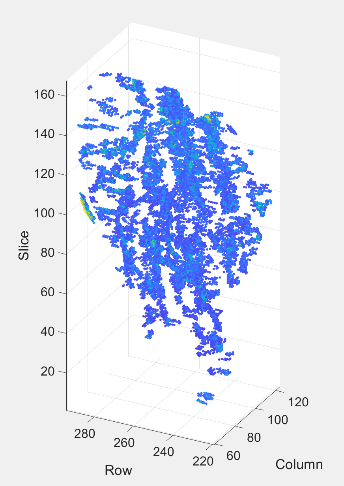

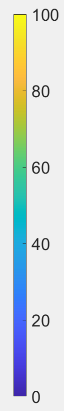


S10


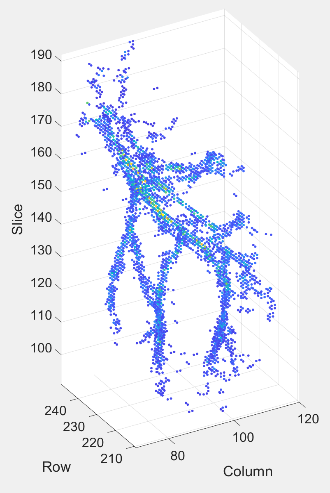

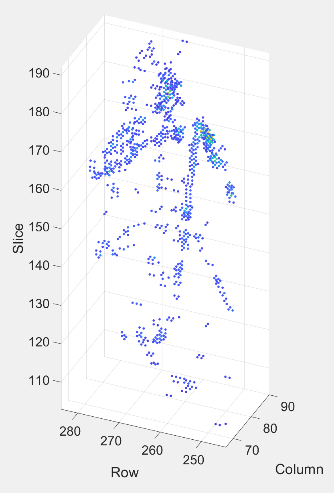

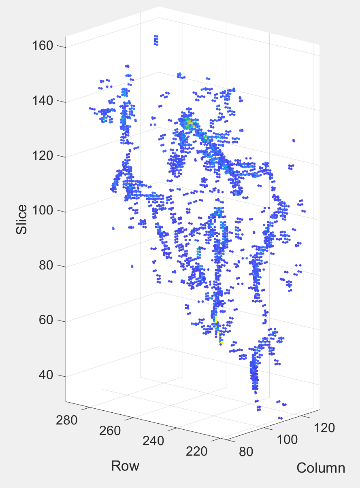

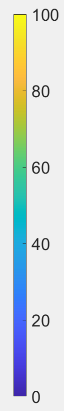


S11


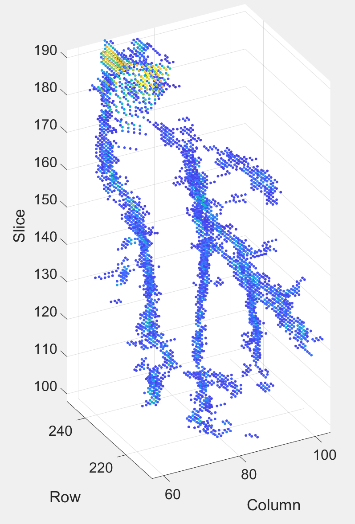

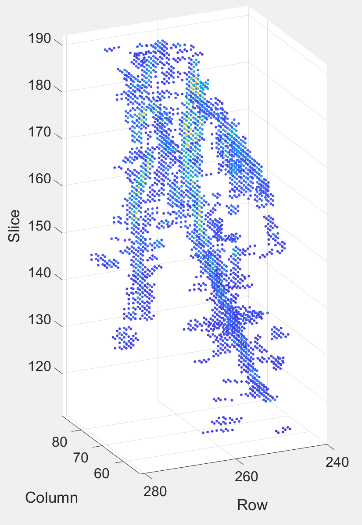

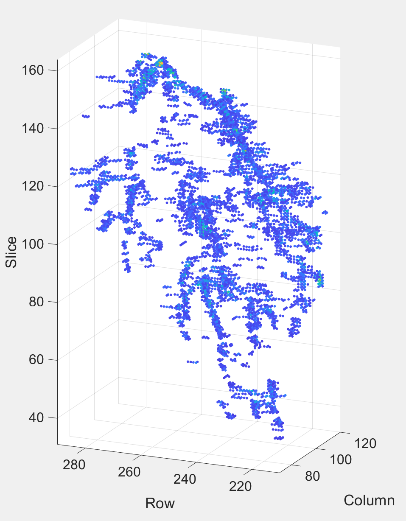

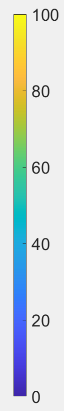


S13


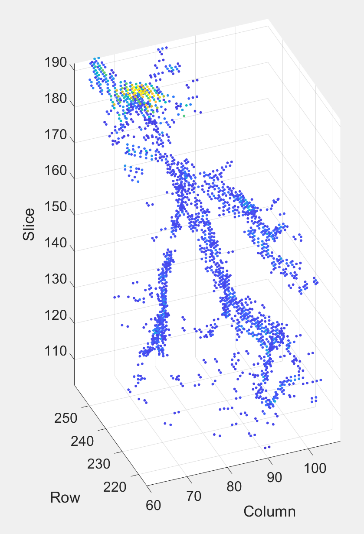

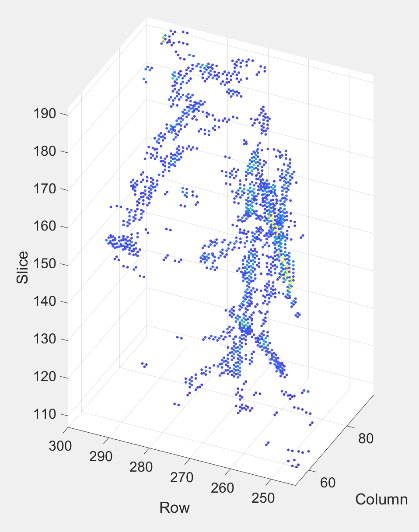

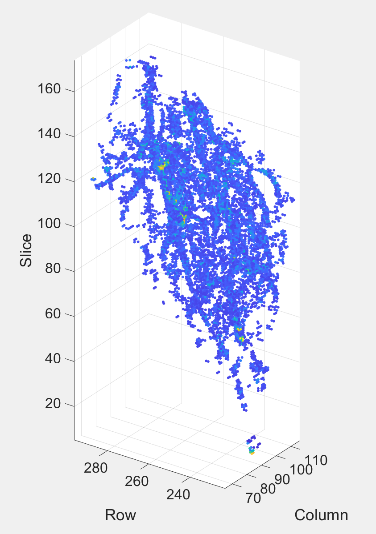

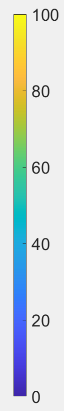


S26


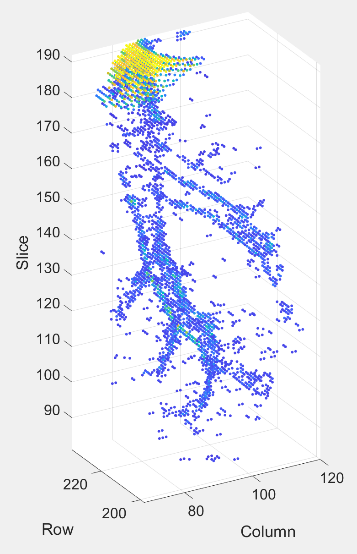

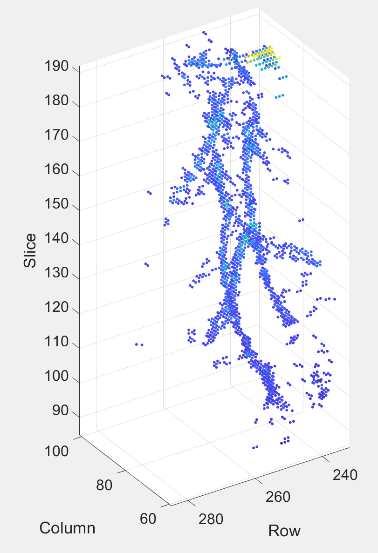

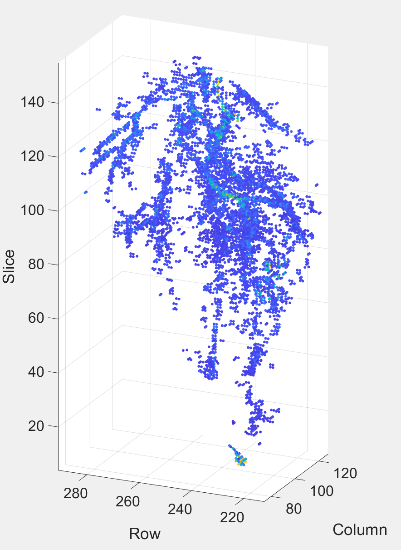

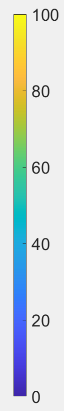


S27


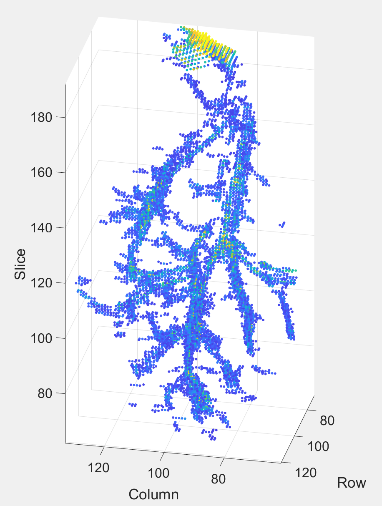

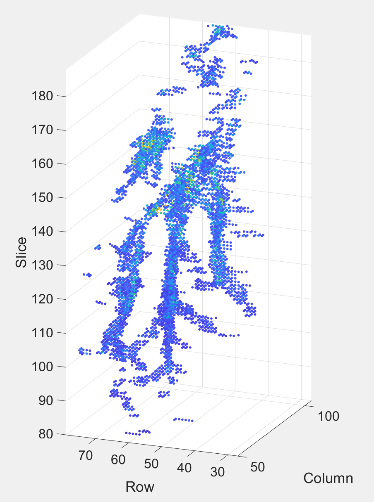

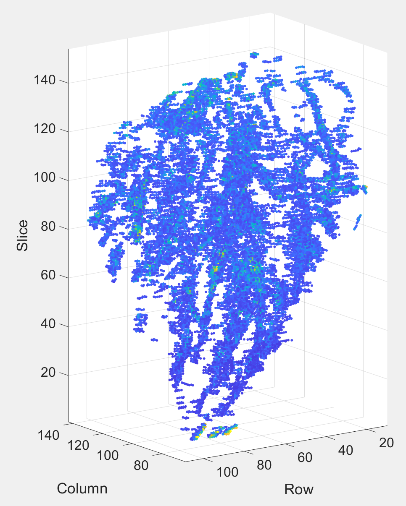

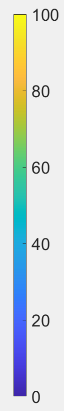


S29


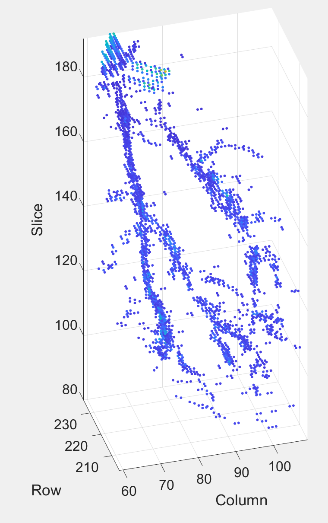

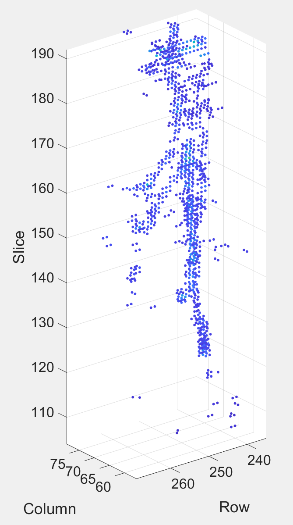

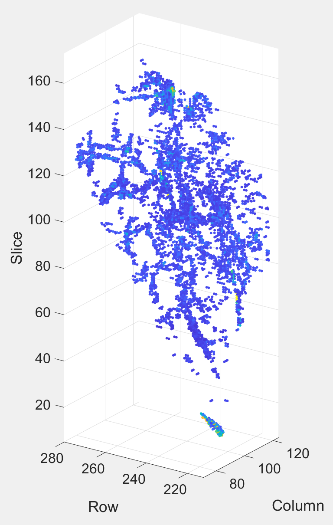

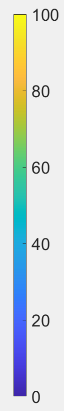


S35


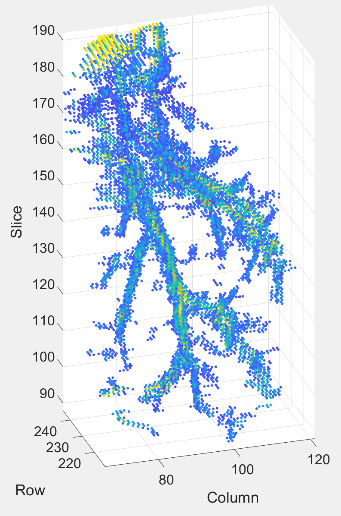

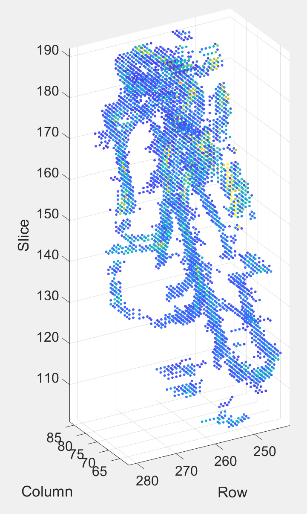

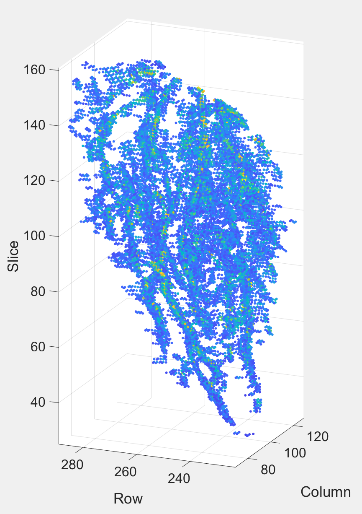

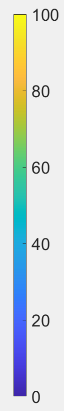


**YF Group (in the order of MG LG and SOL from left to right with participant number on top left)**

S04

**
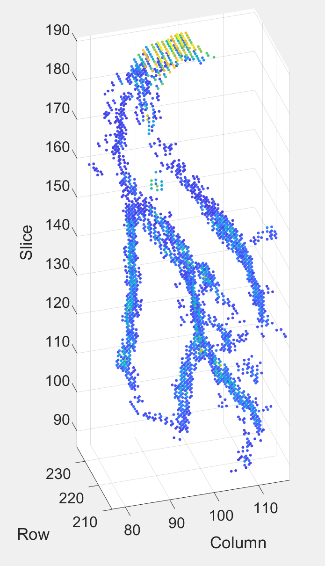
** **
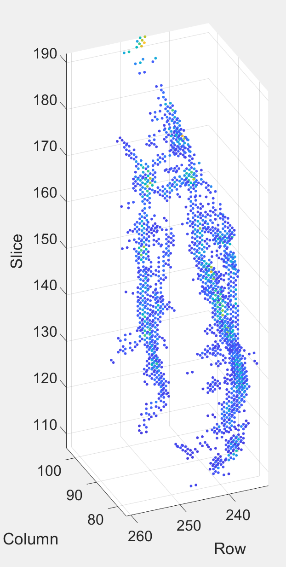
**
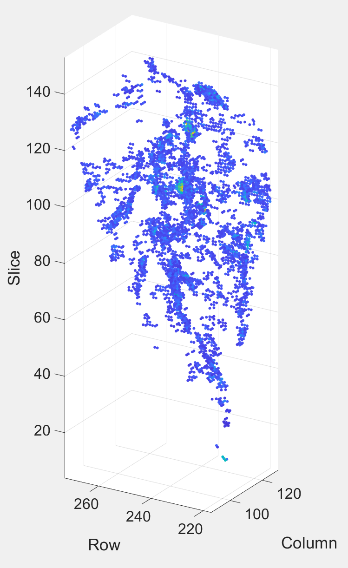

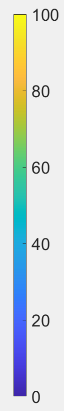


S05

**
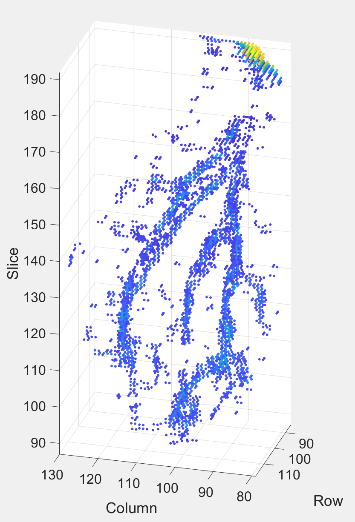

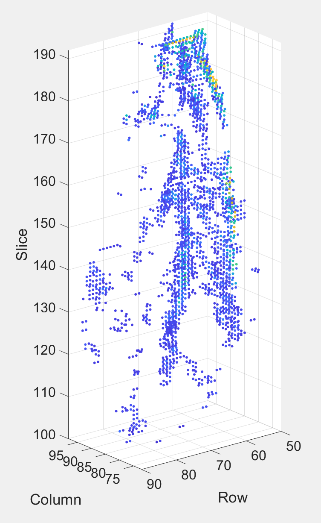

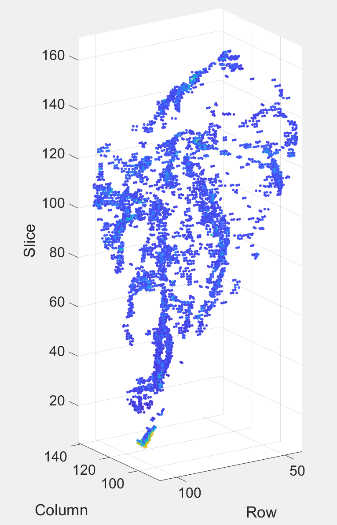
**
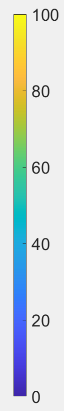


S08

**
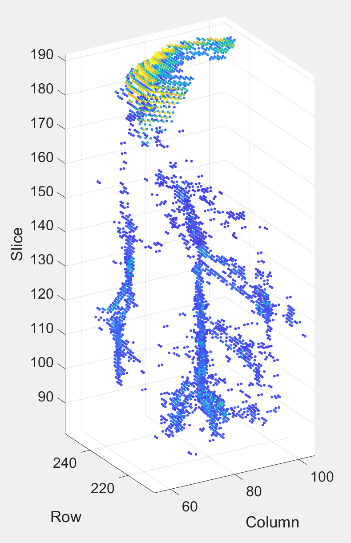

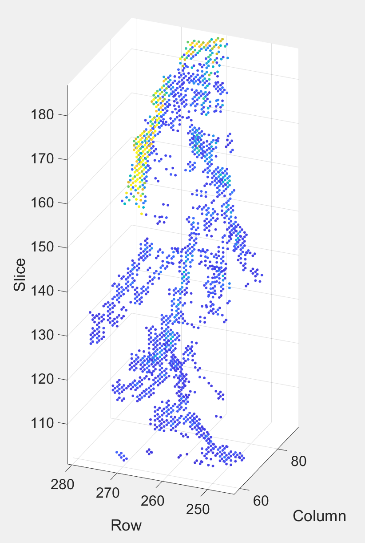

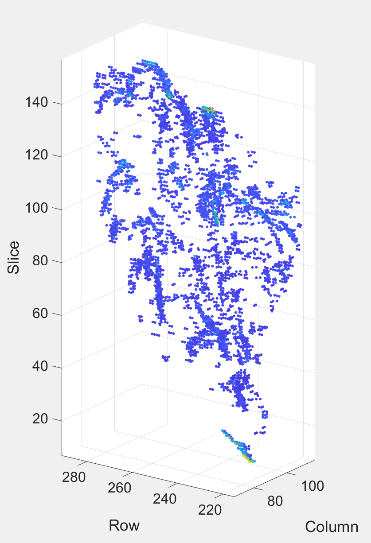
**
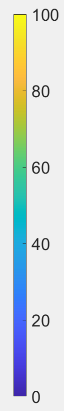


S12


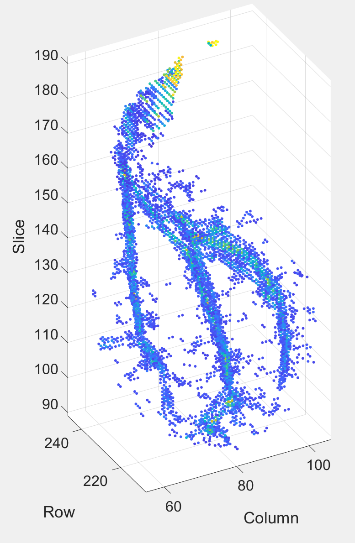

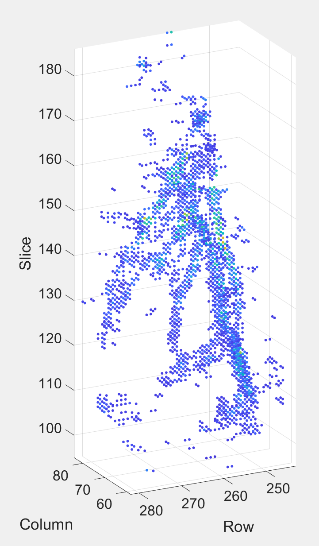

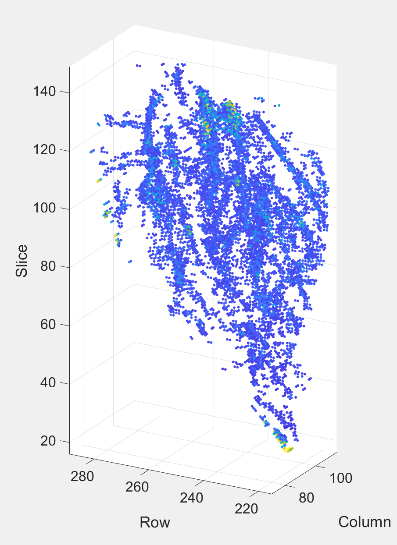

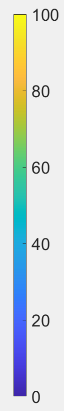


S14


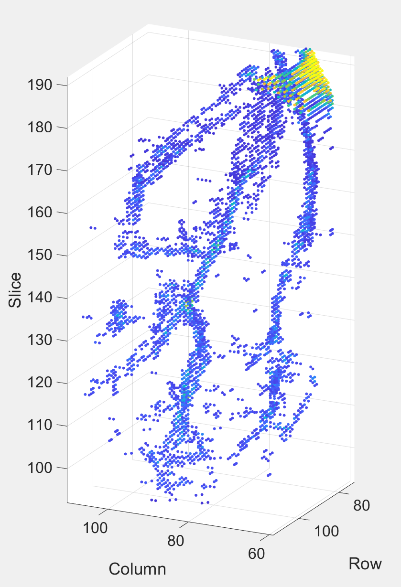

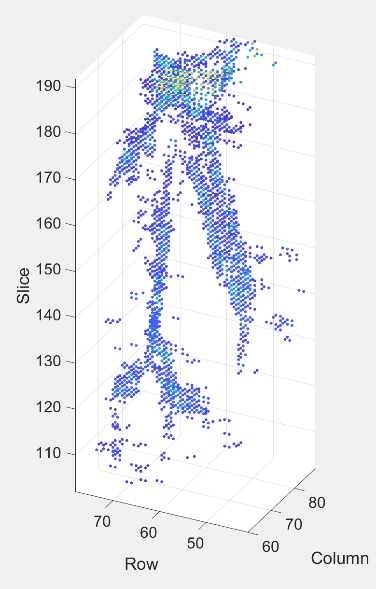

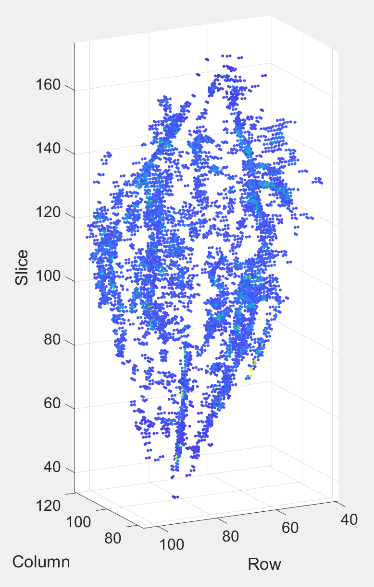

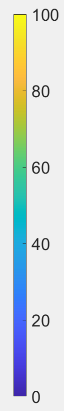


S28


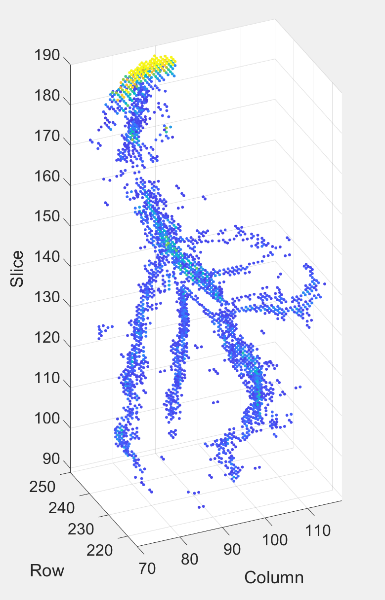

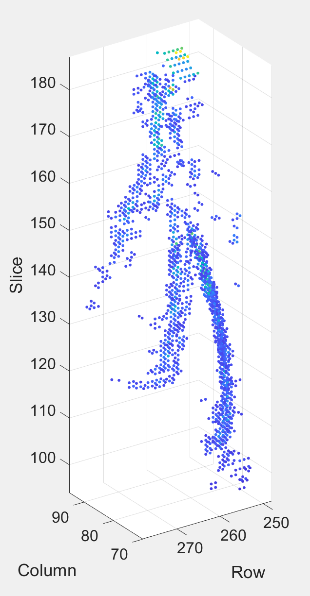

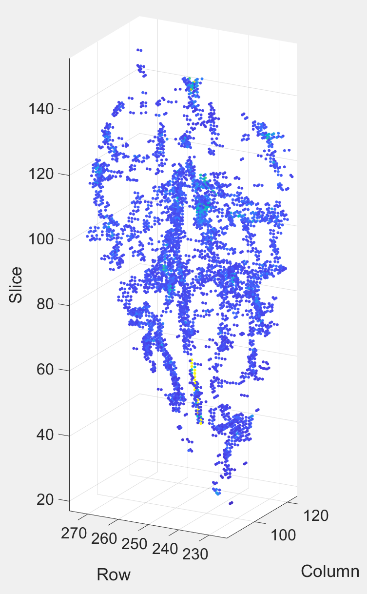

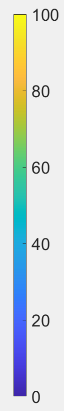


S33


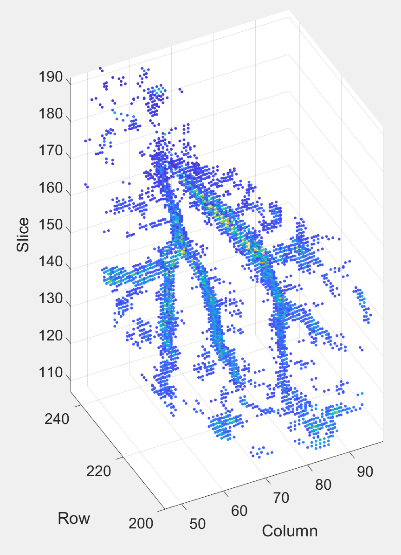

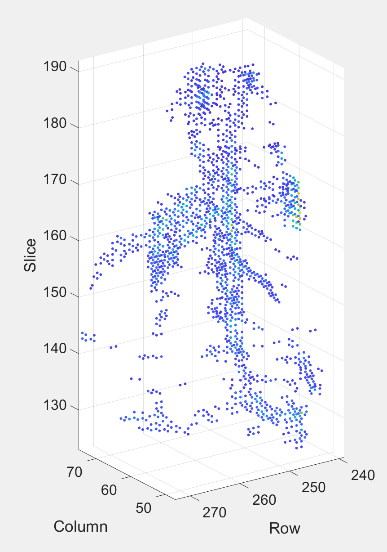

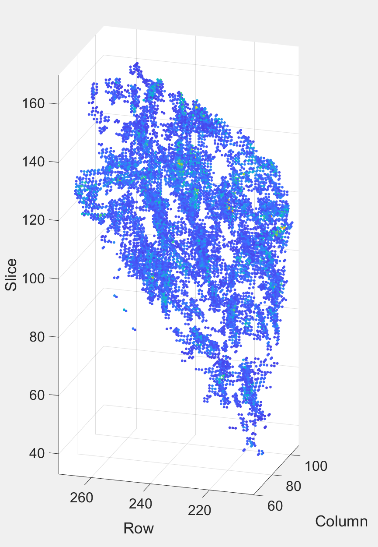

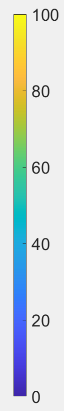


**OM Group (in the order of MG LG and SOL from left to right with participant number on top left)**

S18

**
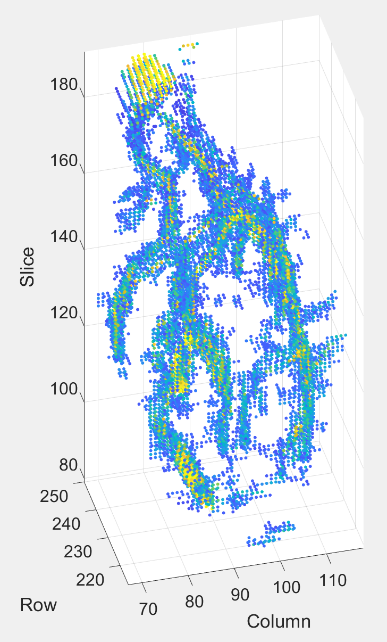
** **
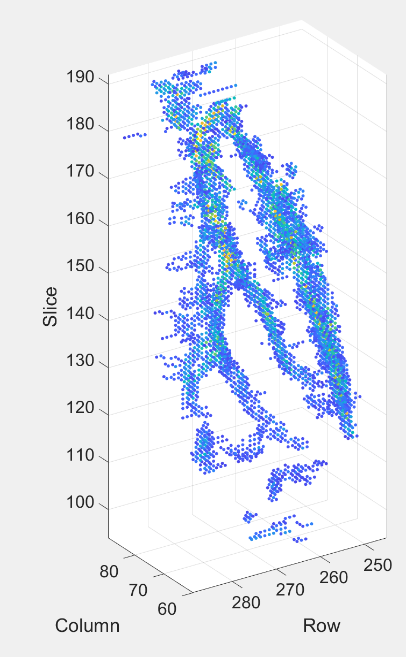
**
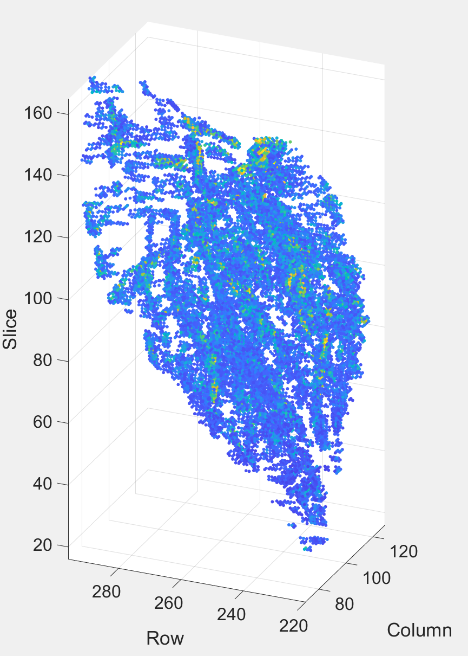

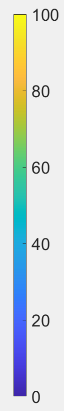


S19

**
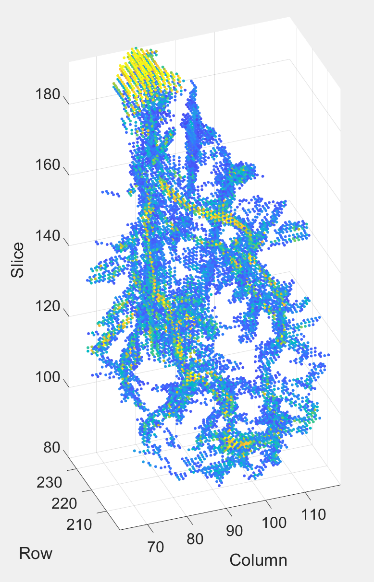

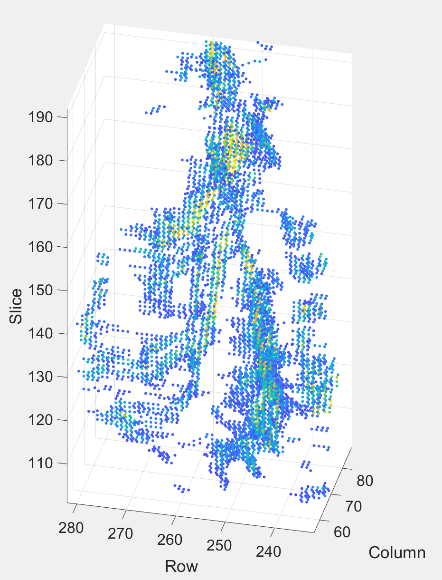

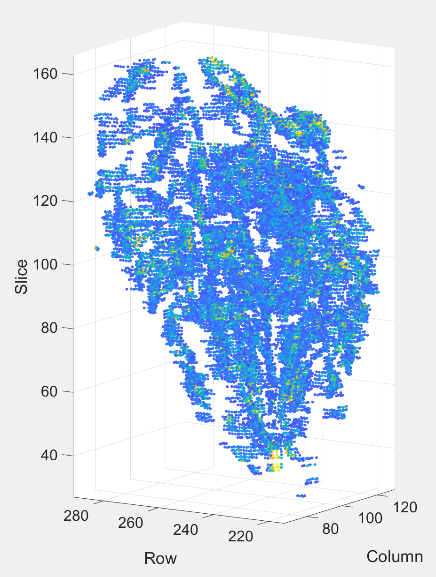
**
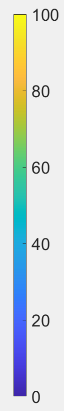


S20

**
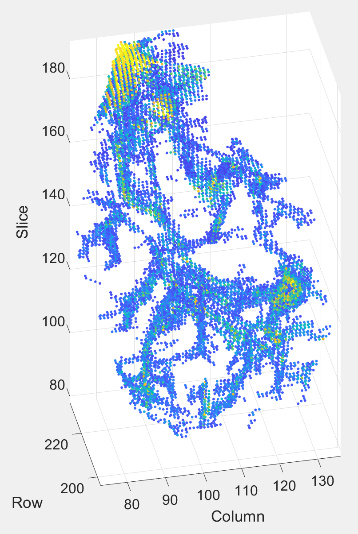

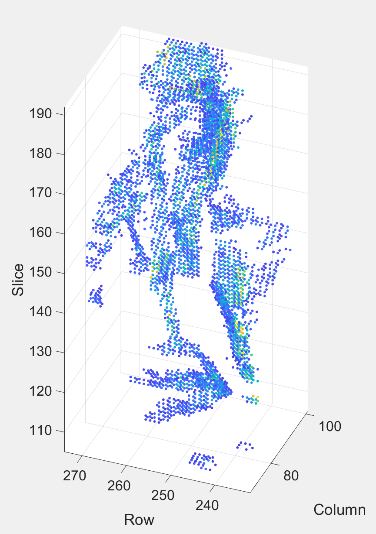

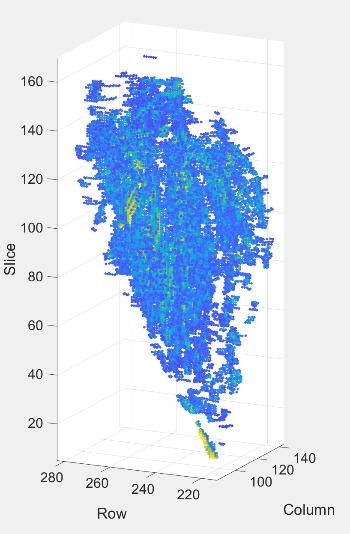
**
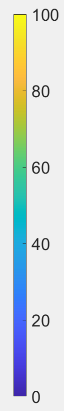


S22


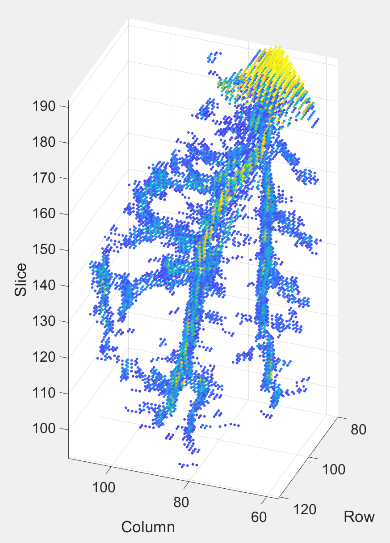

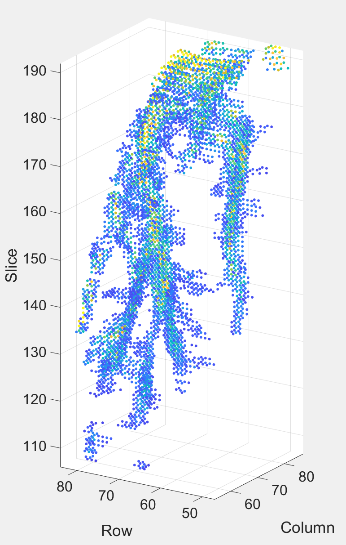

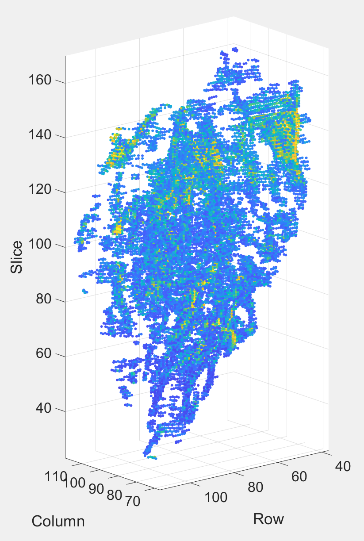

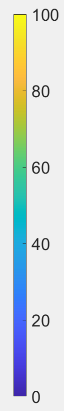


S24


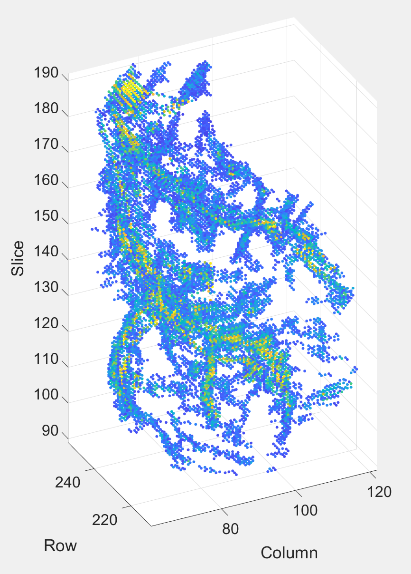

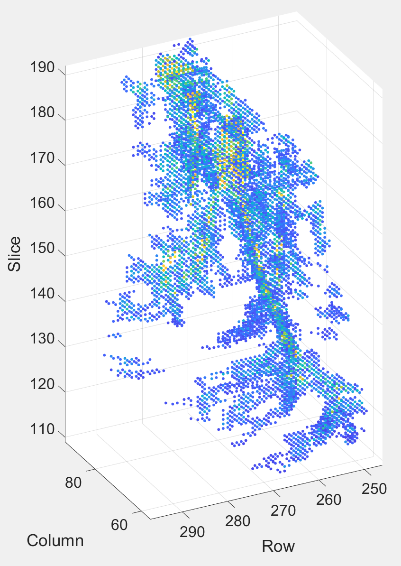

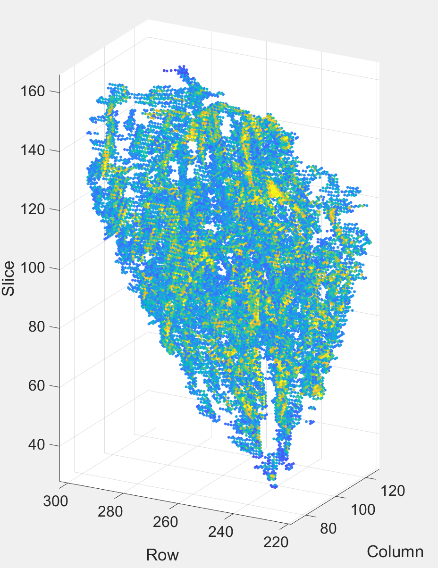

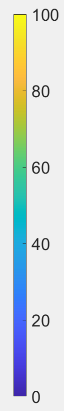


S25


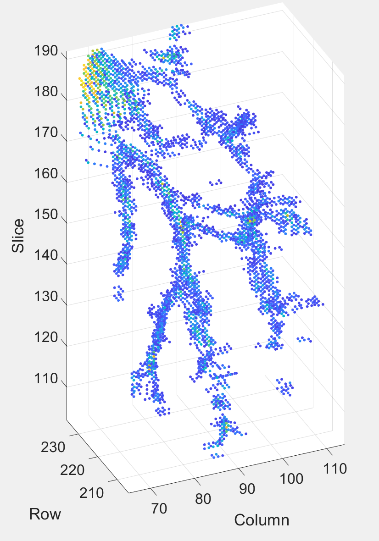

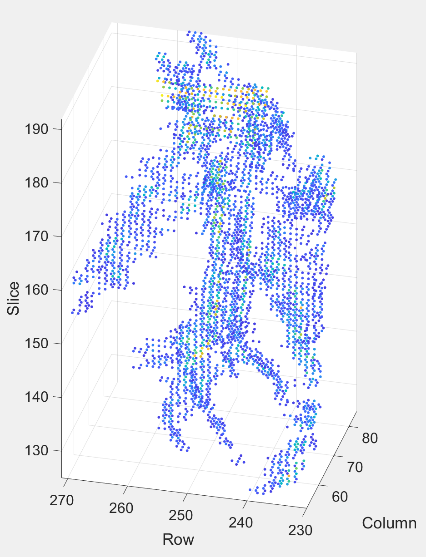

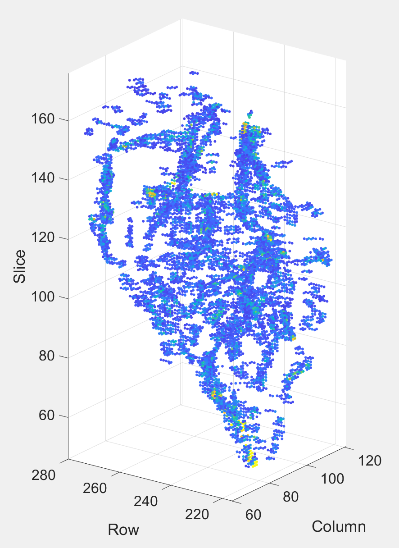

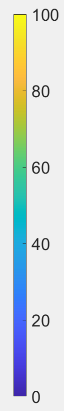


S30

S37

**OF Group (in the order of MG LG and SOL from left to right with participant number on top left)**

S16

S17

S21

S31

S32
